# A novel Tc17 population recruited by tumor cells promotes tumor progression in gastric cancer

**DOI:** 10.3389/fonc.2025.1592328

**Published:** 2025-05-16

**Authors:** Yichao Wang, Hao Yuan, Yayun Tan, Li Wu, Xinchen Zhao, Zeyuan Yang, Shaoxing Dai, Naixue Yang

**Affiliations:** ^1^ State Key Laboratory of Primate Biomedical Research, Institute of Primate Translational Medicine, Kunming University of Science and Technology, Kunming, Yunnan, China; ^2^ Yunnan Key Laboratory of Primate Biomedical Research, Kunming, Yunnan, China; ^3^ Department of Basic Medical Sciences, Tsinghua University School of Medicine, Beijing, China; ^4^ School of Medicine, Nankai University, Tianjin, China

**Keywords:** gastric cancer, tumor microenvironment, single-cell RNA-seq, Tc17 cells, CXCL16

## Abstract

**Objective:**

The tumor microenvironment (TME) plays a crucial role in tumor progression. Recent advancements in single-cell sequencing technology have identified Tc17 cells, a newfound subset of CD8^+^ T cells with a phenotype similar to Th17, within the gastric cancer (GC) microenvironment. However, the role of Tc17 cells are unclear.

**Materials and method:**

We collected 296,879 cells from the single-cell sequencing data of 65 GC samples, along with spatial transcriptomic data from 10 GC samples, to further explore the role of Tc17 cells. Multicolor immunohistochemical staining demonstrated the presence of Tc17 cells in the GC TME. SCENIC analysis was performed to identify the specific transcription factors of Tc17. Pseudotime analysis of T cells was conducted to decipher the differentiation trajectory of Tc17. Additionally, cell-cell interaction analysis revealed the interactions between Tc17 cells and tumor cells. Finally, functional validation through CCK-8 proliferation assays, wound Healing assays, and transwell assays conclusively demonstrated the tumor-promoting effects of IL-17A and IL-26 on gastric tumor cells.

**Results:**

Our results indicate that Tc17 cells are absent from blood and exclusively found in tissues, with greater enrichment in tumor tissues compared to normal tissues. Among CD8^+^ T cells, Tc17 exhibits the weakest cytotoxic capacity and the highest anti-inflammatory profile, suggesting reduced tumor-killing ability. Pseudotime analysis revealed that Tc17 cells ultimately progress towards a state of exhaustion, losing their capacity to eliminate tumor cells. Survival analysis further correlates the presence of Tc17 cells with poor prognosis. Notably, a robust interaction between Tc17 cells and tumor cells was observed in GC. We found that tumor cells can recruit Tc17 cells via the CXCL16-CXCR6 axis, this finding was validated on spatial transcriptome data. And in turn, Tc17 cells may promote tumor progression by secreting IL-17A and IL-26, as functionally corroborated by CCK-8 Assay, wound healing, and transwell assay *in vitro*.

**Conclusions:**

These findings reveal the immunosuppressive role of Tc17 cells in gastric cancer, provide a new perspective for understanding the immunosuppressive mechanism of the gastric cancer tumor microenvironment, and provide a potential target for the development of targeted therapeutic strategies

## Graphical Abstract

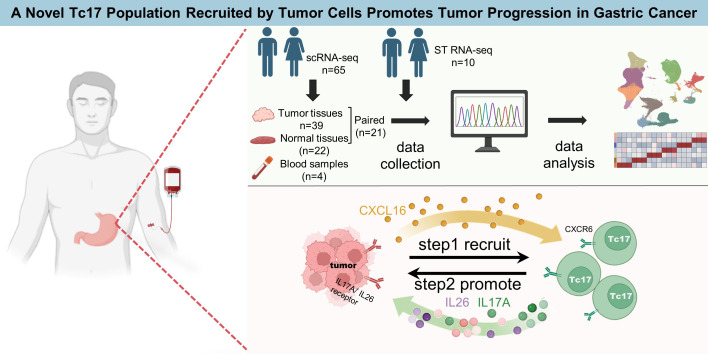

## Introduction

GC is the fourth leading cause of cancer-related mortality globally and the fifth most frequently diagnosed cancer, characterized by a generally poor prognosis (1). The etiology of GC is complex and multifaceted, involving a variety of factors such as viral infections, tobacco use, and a high-salt diet, with Helicobacter pylori infection being the most significant contributor (2, 3). Current treatments, including neoadjuvant chemotherapy, radiotherapy, and molecular targeted therapies, are often hindered by challenges in early detection and the low tolerance of perigastric organs to discomfort (3, 4). Advances in immunotherapy-such as immune checkpoint inhibitors (ICB), chimeric antigen receptor chimeric antigen receptor (CAR) -T cell JYYPtherapy, and tumor vaccines- have demonstrated promising outcomes across various cancer types. ICBs target key regulatory proteins such as CTLA-4, PD-1, and PD-L1, stimulating an enhanced immune response against tumors. ICBs have become a new standard of targeted therapy for advanced or metastatic GC. Clinical studies indicate that the combination of nivolumab and chemotherapy in first-line therapy improves overall survival (OS) in PD-L1-positive GC patients, with approvals in Europe, the USA, and Taiwan (5). Despite these significant strides, the efficacy of immunotherapy in GC remains limited by the immunosuppressive TME, low tumor mutational burden and few neoantigens for immunotherapy to target, and high heterogeneity (4, 6). Therefore, a more profound comprehension of the GC TME is imperative for discovering novel therapeutic targets to improve immunotherapy outcomes.

The emergence of single-cell technology in recent years has provided novel opportunities for the exploration of the GC microenvironment. Notably, Sun et al. had contributed a detailed single-cell transcriptomic landscape of GC, revealing the molecular characteristics and intercellular communication within immune and stromal compartments (7). Kumar et al. discovered an enriched plasma cell presence in diffuse GC, proposing the INHBA and TGFβ superfamilies as potential therapeutic targets for modulating cancer-associated fibroblasts (8). Previous studies revealed five distinct tumor cell subgroups, indicating that low differentiation is predictive of poor prognosis in GC. These identified subgroups with differentiation grades corresponded to Lauren’s histopathological subtypes, including a novel subgroup characterized by immune-related gene expression signatures suggestive of Epstein-Barr virus involvement (9). Additional research has reinforced the association between poorly differentiated GC and the epithelial-mesenchymal transition program, which is often linked to low immune activity (10). However, single-cell omics studies of GC TME face numerous limitations, including challenges in sample collection, inadequate sequencing depth, and variable RNA quality, along with a lack of identified potential therapeutic targets.

Th17 cells, a subset of CD4^+^ T cells, are characterized by the production of cytokines, including interleukin-17 (IL-17), IL-21, and IL-22. Th17 cells contribute to the orchestration of inflammation and tissue repair mechanisms by inducing stromal cells to secrete various cytokines (11, 12). They exert a significant influence on the initiation and progression of a range of inflammatory conditions and metastatic spread of tumors (13–18). Tc17 cells, the CD8^+^ T cell counterpart of Th17 cells, share similar phenotypes and are driven by the same cytokines with Th17 cells polarization, including IL-23, IL-21 in combination with TGF-β1, or IL-6 (19). Within the GC TME, Tc17 cells have been characterized as exhibiting features of tissue-resident memory T cells, with a tendency to differentiate into an exhausted phenotype, highlighting their dynamic role (7). Previous studies have found that Tc17 cells can induce tumor cells to promote the accumulation of myeloid-derived suppressor cells (MDSCs), thereby creating an immunosuppressive microenvironment in GC (20). While the presence and differentiation of Tc17 cells in the GC TME have been partially described, there is currently insufficient data to confirm their unique characteristics or their interactions with tumor cells. Elucidating this relationship is essential for the identification of novel therapeutic targets.

In this study, we integrated data from existing sc-RNAseq data of GC, including GC tumor tissue, adjacent normal tissue, and peripheral blood. Additionally, we collected spatial transcriptomic data from 10 GC samples to further validate our findings. Our results showed that Tc17 cells enriched in tumor tissues lose their tumor-killing ability and tend to reach an exhausted and dysfunctional state. Cell-cell communication analysis demonstrated a strong interaction between Tc17 cells and tumor cells, where tumor cells recruit Tc17 cells via the CXCL16-CXCR6 axis. In turn, Tc17 cells may promote tumor progression by secreting IL-17A and IL-26. Survival analysis further confirmed that the presence of Tc17 and its secreted cytokines are closely related to poor prognosis. These findings provide valuable insights into the mechanisms underlying the actions of the newly found Tc17 cells and the formation of an immunosuppressive microenvironment in GC, suggesting that targeting Tc17 cells could be a promising therapeutic strategy for GC.

## Materials and methods

### Single-cell RNA-sequencing data process

ScRNA-sea data are download from GEO (Access numbers: GSE183904) and the Genome Sequence Archive in BIG Data Center, Beijing Institute of Genomics (BIG), Chinese Academy of Sciences (Access numbers: HRA000704). We use the “merge” function to merge the two batches of data, and then use the R package “Seurat” (version 4.2.0) for subsequent analysis. Initially, We have performed strict quality control, genes expressed in less than three cells were filtered out, cells with either fewer than 501, over 7000 UMIs or mitochondrial ratio is higher than 20 were filtered out. Subsequently, we use the R package “*DoubletFinder*” (version 2.0.3) to remove any double cells that might be present in each sample. Finally, a comprehensive global scaling normalization procedure was executed using the “*NormalizeData*” function, employing the “*LogNormalize*” method for each sample. The identification of the top 10000 highly variable genes were carried out using the “*FindVariableFeatures*” functionality. To integrate cells from different samples, we used the “*FindIntegrationAnchors*” function to identify “anchors” between samples and then integrated the datasets with the “*IntegrateData*” function with 30 dimensions. This returned a Seurat object with an integrated expression matrix for all nuclei. We scaled the integrated data with the “*ScaleData*” function. Subsequently, principal component analysis (PCA) dimensionality reduction was employed via the “*RunPCA*” method. In parallel, the Uniform Manifold Approximation and Projection (UMAP) technique was executed, yielding a compacted representation of the data within a reduced-dimensional space. The clustering procedure was carried out through the “*FindNeighbors*” and “*FindClusters*” functions, which employed a shared nearest neighbor (SNN) modularity optimization-based clustering algorithm to delineate distinct cell clusters.

### Description of T cell subpopulations

CD8_Naive and CD4_Naive were in a naïve state (expressing LEF1 and SELL). The clusters CD8_GZMK_ZNF683 and CD8_Tem were noted for their high expression of cytotoxic genes (GZMK, GZMH). The CD8_Tem cluster was defined as effector memory cells, characterized by elevated expression levels of GZMK, CD44, and CXCR4. Tissue-resident memory CD8^+^ T cells (CD8_Trm) were labeled by the expression of TOB1 and ANXA1. Mucosal-associated invariant T cells (MAITs) expressed SLC4A10 and RORA. CD8_Blood_Teff in blood and CD8_Teff cells exhibited expression of genes associated with effector functions(FCGR3A andGNLY). Intraepithelial lymphocytes (CD8_IEL) expressed natural killer cell marker genes (KLRC1 and CD160). The CD4_Blood_Tcm and CD4_Tcm were blood central memory cells(S1PR1 and ICAM2), and tissue central memory cells (TCF7 and GPR183). CD4_Activated exhibited a distinct activation state (CD69). CD8_Exhausted and CD4_Exhausted expressed immune checkpoint genes such as CTLA4, PDCD1, and TIGIT. Treg_IL10, Treg_Suppressive, and Treg_Naive represent distinct Treg subtypes, all of which express CD4 and FOXP3. However, each subtype exhibits unique marker expression profiles: Treg_Naive expressed naïve markers (LEF1, SELL), Treg_Suppressive expressed suppressive markers (CTLA4), and Treg_IL10 expressed regulatory T cell markers (IL10). Interestingly, we observed clusters expressing IL-17A in both CD4^+^ and CD8^+^ T cells, which we designated as Th17 and Tc17, respectively.

### Quantification of tissue enrichment analysis

To evaluate the tissue preference of each cell cluster, we calculated the ratio of observed to expected cell numbers in each cell cluster across different tissues to get the ratio of observation to expectation (*Ro/e*). For a given cell cluster, *Ro/e* > 1 suggests that this cluster is enriched in a specific tissue, and *Ro/e* < 1 suggests that this cluster is depleted in a specific tissue.

### Gene sets score analysis

The “Cytolytics effector pathway” and “Anti-inflammatory” gene sets are presented in the supplementary file. The signature score of gene sets were calculated using the “*AddModuleScore*” function in the “Seurat” R package with default parameters.

### Pseudotime analysis

Pseudotime analysis was performed on T cells with the “Monocle3” R package (version 1.0.0) (Cao et al, 2019). We map the results of the UMAP dimensionality reduction cluster to the results of the monocle3 cluster,and then, the trajectory analysis was performed using the “*learn_graph*” function, cell ordering using the “*get_earliest_principal_node*” function. DEGs along the pseudotime trajectory were identified by the “*choose_cells*” and “*graph_test*” functions of Monocle3 with the cutoff of q-value < 0.05.

### Deconvolution

We downsampled the expression matrix, sampling 100 cells per cell type, and used the “*Create Signature Matrix*” module from the online CIBERSORTx portal (https://CIBERSORTx.stanford.edu) to generate the signature matrix. Subsequently, we used the “*Impute Cell Fractions*” module with a Run mode “*absolute*” and Permutations of 500 to deconvolution predict the proportion of cells in Bulk dataset.

### Identification differentially expressed genes

To identify genes specific to Tc17 compared to Tex or Th17, we used the “*FindMarkers*” function in Seurat. Only genes with adjusted p-value < 0.01(Wilcoxon test) were selected. In order to verify the classification of tumor cells, we collected TCGA dataset to observe the correlation between the differential genes in the tumor samples and the normal samples. We calculated the log2FC values respectively and selected the top 1000 genes for Spearman correlation. In single-cell data, we also used the “*FindMarkers*” function to find differentially identified genes between tumor cells and normal cells, with adjusted p-value < 0.01 and log2FC > 1 was identified as a tumor up-regulated gene and adjusted p-value < 0.01 and log2FC < -1 was identified as a down-regulated gene. Subsequently, these genes were selected for further enrichment analysis.

### Cell-cell communication analysis

For a comprehensive analysis of cell-cell communications, “iTALK” (version:0.1.0) was used to identify number of potential ligand-receptor pairs between cell types. The top 50 percent highly expressed genes were used to find ligand-receptor pairs. iTALK annotates ligand-receptor pairs into 4 categories: cytokines, growth factors, immune checkpoints and others. Next, “CellChat” (version:2.1.2) with default parameters was used to identify potential ligand-receptor pairs between cell types. CellChat is an analytical tool that accepts gene expression data as user input to model the likelihood of cell-to-cell communication. It integrates this data with an existing database that catalogues known interactions between signaling ligands, receptors, and their cofactors, providing a comprehensive assessment of the potential for cellular interaction. After annotating the objects with relevant labels and identifying the differentially expressed genes, the “*computeCommunProb*” function was used to infer the communication probabilities between cells, and then utilize the “*filterCommunication*” function to eliminate cell-cell communication events that involve cells with expression levels below the threshold of 3, thereby refining the analysis to include only those cells that demonstrate significant and consistent gene expression. The “*computeCommunProbPathway*” function was used to generate intercellular communication for each cell signaling pathway. Ultimately, we employ the function “*aggregateNet*” to compute the aggregated network, which quantifies the cell-cell interactions by tallying the number of connections or by summarizing the probabilities of communication.

### The activity of transcription factor regulon

Transcription factor regulon activity was assessed using “pySCENIC” (version 1.0.1) (21). To briefly explain the process, regulons were initially identified by examining the coexpression patterns between transcription factors and their target genes. This was followed by a motif analysis to confirm the regulatory interactions. The activity of each regulon in individual cells was quantified using the AUCell algorithm, which computes a score between 0 and 1, indicating the regulon’s activity level. We then calculated the average activity value of each regulon in a certain group of cells and scale these values to obtain a specific regulon for each cell type.

### Survival analysis

We grouped the different proportions of Tc17 cells in the cibersortx predicted TCGA dataset, and then, we used the R package “survival” (version:3.7.0) to perform survival analysis. Furthermore, we performed survival analysis of CXCL16 gene expression in tumor cells using the “Survival Analysis” module of the online portal GEPIA2 (http://gepia2.cancer-pku.cn/#survival).

### Copy number alteration prediction

Selecting a reference cell cluster, T&NK cells were chosen as a reference for this analysis, we utilized the infercnv package (version:1.18.1) (InferCNV of the Trinity CTAT Project: https://github.com/broadinstitute/InferCNV) to infer copy number variations in cells, thus identifying malignant cell clusters.

### Immunofluorescence staining

During paraffin combined with Immunofluorescence, a series of rigorous dewaxing and hydration steps are first performed on the gastric cancer sections. The sections are sequentially immersed in xylene I for 10 minutes and xylene II for another 10 minutes to completely remove the paraffin. Subsequently, the sections are soaked in anhydrous ethanol I and anhydrous ethanol II for 7 minutes each, followed by immersion in 95%, 85%, and 75% alcohol solutions for 5 minutes each to gradually hydrate the tissue. Finally, the sections are washed with distilled water for 5 minutes to ensure a clean surface free of impurities, laying a solid foundation for subsequent steps. For antigen retrieval and blocking, a histochemical pen is used to carefully draw a blocking circle around the gastric cancer tissue to ensure that subsequent reagents act only on the target area. An appropriate amount of pepsin is then added within the circle to fully cover the tissue, ensuring adequate exposure of antigen sites. The sections are incubated in a 37°C oven for 30 minutes to allow thorough antigen retrieval. After incubation, the sections are thoroughly rinsed with PBS buffer to remove residual pepsin. The slides are then placed in PBS (pH 7.4) and gently agitated on a shaker at an appropriate speed for three washes, each lasting 7 minutes, to further remove impurities and ensure the cleanliness of the section surface. primary antibodies overnight at 4°C and fluorescence-labeled secondary antibodies at room temperature for 1 h. Counterstained with Hoechst 33342 (Thermo Fisher, H3570, 1:1000) to visualize the nuclei. Theslides were then examined using a Leica TCS SP8DIVEconfocal microscope. The antibodies used for immunofluorescence staining in this study are as follows: anti-CD8(Servicebio, GB12068, 1:1000), anti-KRT17(Servicebio, GB15363, 1:300), anti- RORC (Servicebio, R50198, 1:200). Secondary antibodies used were the following: goat anti-mouse AF488 (Servicebio, GB23301, 1:500), goat anti-mouse AF555 (Servicebio, GB23301, 1:500), goat anti rrabbit-AF647 (Servicebio, GB23303, 1:500).

### CCK8 assay

To evaluate the effects of interleukins on gastric cancer cell proliferation, a CCK8 colorimetric assay (Mei5bio, Cat# MF128-01) was performed using HGC-27 cells (Pricella, Cat# CL-0107). Cells were seeded in 96-well plates at a density of 1×10^4^ cells/well and divided into four groups: 1) untreated control (CT), 2) IL-17A (50 μg/ml), 3) IL-26 (50 μg/ml), and 4) IL-17A+IL-26 co-treatment (50 μg/ml each). After 24 h of adherent culture, the original medium was replaced with 100 μl fresh medium containing 10 μl CCK-8 reagent per well. Following 2 h incubation at 37°C, absorbance at 450 nm was measured using a multimode microplate reader (Thermo Scientific™ FC). All groups contained triplicate wells, with three independent biological replicates.

### Wound healing

To evaluate the impact of the identified interleukins on the migratory capacity of tumor cells, we performed the scratch migration assay. The specific experimental protocol is as follows: Gastric cancer cells HGC-27 were seeded at a density of 5×10^5^ cells per well in 6-well plates pre-coated with scratch assay-specific culture membranes (Beyotime, Cat# FAM106-80). The cells were routinely cultured until the confluence reached 90%–100%. The scratch assay-specific culture membranes were gently removed, and the wells were rinsed three times with PBS to remove detached cells. The cells were divided into four treatment groups: 1) Blank control group (CT); 2) IL-17A treatment group (50 μg/ml); 3) IL-26 treatment group (50 μg/ml); 4) Combined IL-17A and IL-26 treatment group (each at 50 μg/ml). Fresh serum-free culture medium was added to maintain a consistent volume across all groups, and the cells were incubated in a 37°C, 5% CO^2^ incubator. Images of the same scratch area were captured using an inverted microscope at 0 h and 24 h, respectively.

### Transwell invasion assay

We assessed the effects of IL-17A and IL-26 on the invasive capacity of gastric cancer cells using the Transwell invasion assay. The experiment utilized Transwell inserts with an 8-μm pore size, which were coated with Matrigel matrix (Beyotime, Cat# C0372-1ml). Gastric cancer cells were first subjected to serum starvation for 24 hours. A total of 2×10^5^ gastric cancer cells were then seeded into the upper chamber and treated with 50 μg/ml IL-17A, IL-26, or a combination of both. The lower chamber was filled with culture medium containing 20% fetal bovine serum as a chemoattractant. After 24 hours, the cells were fixed and stained.

## Results

### The single-cell landscape of gastric cancer microenvironment

**Figure 1:**
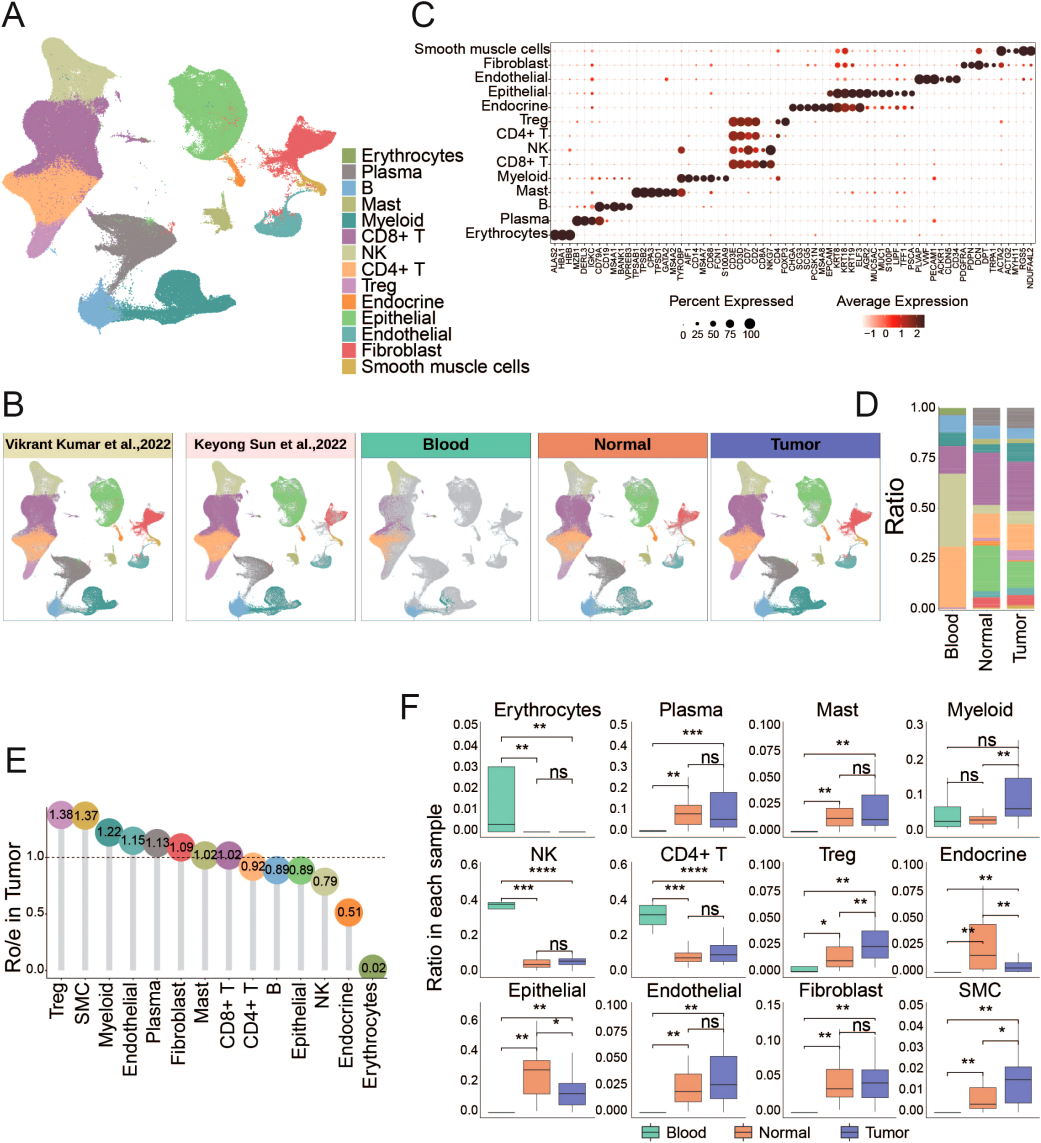
Overall landscape of gastric cancer microenvironment **(A,B)** The UMAP shows the distribution of different cell types, different data sources, and different tissue sources. The number of cells in different populations is: Erythrocytes (795), plasma (26426), B (17605), mast (6381), Myeloid (22617),CD8^+^ T (71534), NK (24572), CD4^+^ T (41994), Treg (11123), Endocrine (2884), Epithelial (44066), Endothelial (9323), Fibroblast (14094), smooth muscle cells (3465). **(C)** Dotplots displayed the smoothed expression distribution of marker genes in distinct cell types. Dot size represents the proportion of expressing cells. Color indicates the Z score scaled gene expression levels. **(D)** The bar chart shows the proportion of different cell types in each tissue source. **(E)** Lollipop plot showing the distribution of individual cell types by Ro/e score in GC. Tumor-enriched subpopulations (above 1) and normal-enriched subpopulations (below 1) are highlighted according to Ro/e score. **(F)** The box plot shows statistically significant differences in the proportion of each cell type between different tissues. P-values were calculated by Wilcoxon rank-sum test. *P < 0.05, **P < 0.01, ***P < 0.001, ****P < 0.0001. ns, not significant.

To profile the landscape of GC TME at the single-cell level, we collected scRNA-seq data on tumor tissues, adjacent normal tissues and peripheral blood from 65 samples (7, 8). After strict quality filtering, we collected a total number of 296,879 high-quality cells for subsequent analyses (**Figure 1A**, **Supplementary Figure S1A** and **Supplementary Table S1**). We corrected the batch effect across different samples, and performed non-linear dimensionality reduction and unsupervised clustering, which ultimately identified 14 cell types split into 3 compartments including immune cells (T, B, NK, mast and myeloid cells), stromal cells (fibroblasts, endothelial, and smooth muscle cells), and epithelial cells (**Figure 1A**, **Supplementary Figure S1B**).

The abundance of erythrocytes, CD4**^+^** T cells and NK cells in peripheral blood exceeds that in solid tissues. In contrast, Treg cells, smooth muscle cells, myeloid cells, endothelial cells, plasma cells, fibroblasts cells and mast cells were more abundant in solid tissues (**Figure 1D**, **Supplementary Figure S1C**). The proportion of myeloid cells, Treg cells and smooth muscle cells was significantly increased in tumor tissues compared with adjacent normal tissues (**Figure 1**, **Supplementary Figure S1E**). Myeloid cells, key cellular component of immune cells in TME, play a pivotal role in modulating tumor inflammation and angiogenesis (22). Treg cells are increased in various cancer types such as breast, lung, stomach, and liver cancers, where they contribute to immune suppression by inhibiting T cell proliferation and cytokine production (23–26). SMCs are frequently observed in the vasculature of cancers, forming part of the blood vessel walls. SMCs can exhibit abnormalities in GC, with hypertrophy of the muscularis mucosae and increased thickness of the extratumoral muscularis mucosae. This adverse TME significantly promotes cancer progression and may lead to the failure of cancer treatment (27).

### CD8^+^ IL17A^+^ Tc17 cells were enriched in tumor tissues and associated with poor prognosis

**Figure 2:**
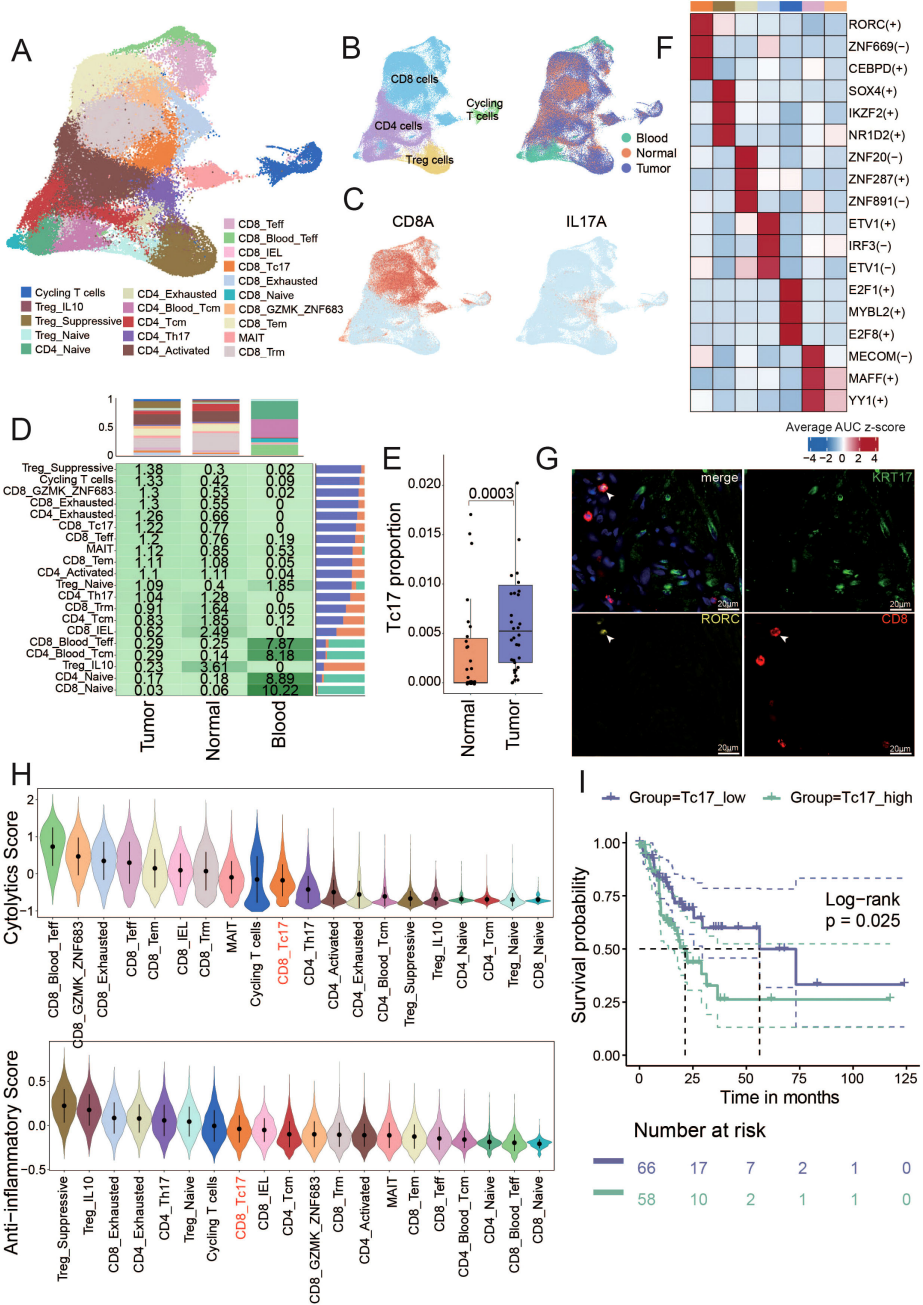
Identification of T cell subsets and unique characteristics of Tc17 cells **(A)** The UMAP shows the distribution of different cell types. The number of cells in different subpopulations is: Cycling T (3,201), Treg_IL10 (665), Treg_Suppressive (9,370), Treg_Naive (1,805), CD4_Exhausted (1,295), CD4_Naive (3,902), CD4_Blood_Tcm (4,252), CD4_Tcm (7,370), CD4_Activated (18,051), CD4_Th17 (2,219), CD8_Naive (749), CD8_GZMK_ZNF683 (4,020), CD8_Tem (12,123), MAIT(4,130), CD8_Trm (19,987), CD8_Teff (3,750), CD8_Blood_Teff (2,512), CD8_IEL (505), CD8_Tc17 (3,059), CD8_Exhausted (3,861). **(B)** The UMAP shows the distribution of different tissue sources (right) and four mian clusters (left). **(C)** The UMAP plot illustrates the expression distribution of the Tc17 cells marker genes: CD8A and IL17A. **(D)** The central figures indicate the distribution of various T cell subtypes according to Ro/e scores. The bar chart at the top shows the proportion of individual cell types from different tissue sources, and the bar chart on the right illustrates the percentage of different tissue sources within each cell type. **(E)** Box plot showing the Tc17 cell proportion in normal and tumor samples from single-cell data. P-values were calculated by Wilcoxon rank-sum test. **(F)** Top 3 regulons enriched in each T cell subtype. The darker the color, the stronger the regulatory impact of the modulator. **(G)** Immunofluorescence staining for RORC and CD8 (Tc17 markers, yellow and red), and KRT17 (tumor cells marker, green) along with DNA visualized by DAPI staining in gastric cancer. Images are representative of a single experiment, with three tumors imaged. KRT17 (Keratin 17) is considered an important tumor marker gene in gastric cancer, related to **Figure 5D**. **(H)** The violin plot illustrates the cytotoxic and anti-inflammatory scores of all T cell subtypes, ranked from highest to lowest. **(I)** Kaplan-Meier curves depict the disease-free survival (DFS) of GC patients stratified by the proportion of Tc17 cells in tumor samples. Patients with a high proportion of Tc17 cells are represented in dark green, while those with a low proportion are depicted in purple. The p-value is calculated using the Log-rank test.

T cells play a pivotal role in the TME and cancer immunotherapy. T cells can recognize and eliminate tumor cells through specific antigen recognition and cytotoxic activity, thereby contributing to antitumor immune responses (28–32). Harnessing the functions of T cells has become a key strategy in cancer immunotherapy to enhance antitumor immunity and improve treatment outcomes. To characterize the function of T cells in GC, T cells were reclustered into 20 sub-clusters, including 10 CD8^+^ clusters, 6 CD4^+^ clusters, 3 Treg clusters and a cluster of T cells in a state of proliferation (cycling T cells) (**Figure 2A**, **Supplementary Figure S2A**). For detailed identification of each T cell subset, please refer to the Methods (Description of T cell Subpopulations section).

We found that T cells in peripheral blood predominantly exist in naïve and memory states. CD4_Tcm, CD8_Trm and CD8_IEL T cells became the mainstream in normal tissues (**Figure 2**, **Supplementary Figure S2A**). The accumulation of these T cells with memory and killing ability indicates that the adjacent normal tissue is still in a state of dominated by anti-tumor immunity. CD8_Exhausted and CD4_Exhausted T cells are significantly enriched in tumor tissues. Previous report has revealed that exhausted CD8^+^ T cells are actually highly proliferative within the TME in a state of dynamic differentiation and high activity, and are likely driving tumor-specific immune responses (33). In addition, Treg cells with immunosuppressive functions also accumulate in the tumors. We categorized all T cell subsets based on their Ro/e scores for clustering, to assess subsets enriched in tumor or normal tissues. The clustering analysis revealed seven cell types enriched in tumor tissues (including CD4_Exhausted, CD8_Teff, CD8_Tc17, Treg_Suppressive, Cycling T cells, CD8_GZMK_ZNF683 and CD8_Exhausted), and five cell types enriched in normal tissues (including CD8_Naive, CD4_Naive, Treg_IL10, CD4_Bood_Tcm, CD8_Blood_Teff). The remaining cell types were found in both tumor and normal tissues (**Supplementary Figure S2**).

Notably, we identified a distinct population of IL-17A-secreting CD8^+^ T cells, termed Tc17 cells, which were significantly enriched in the TME. Although both Tc17 and conventional Th17 cells (IL-17A^+^ CD4^+^ T cells) secrete IL-17A and thus cluster closely in transcriptomic analyses, they are clearly distinguished by their surface marker expression (CD8^+^ for Tc17 cells versus CD4^+^ for Th17 cells), reflecting their fundamentally different lineage identities and functional roles (**Figure 2**). Established single-cell studies have confirmed the presence of Tc17 cells in GC microenvironment (7). Few studies have also detected Tc17 cells in the peripheral blood and cerebrospinal in patients with multiple sclerosis (34, 35), linking this to worse survival in patients with cancer (20, 36–38). However, the function and role of Tc17 during tumor progression remains poorly understood. In this study, we found Tc17 was present only in solid tissues and absent from peripheral blood, with significantly higher levels in tumor tissues compared to normal tissues (**Figure 2**, **Supplementary Figures S2C, D**). By analyzing deconvoluted TCGA cohort data and single-cell data on the proportions of Tc17 cells, Th17 cells, and other CD8^+^ cytotoxic T cells, we observed that both Tc17 and Th17 cells were significantly reduced compared to CD8^+^ cytotoxic T cells (**Supplementary Figure S2**). Using pySCENIC to identify the specific transcription factors of each cell type, we identified that the top three transcription factor regulons specifically expressed in Tc17 cells: RORC, ZNF669, and CEBPD (**Figure 2**, **Supplementary Figures S2F, G**, **Supplementary Table S2**). We also demonstrated the presence of Tc17 cells in the GC TME by multicolor IHC staining (**Figure 2**). RORC is crucial in autoimmune responses and inflammation, and it is a key regulator in the proliferation, metastasis, and chemoresistance in various malignancies. The protein encoded by RORC, RORγt, is a pivotal transcription factor for Th17 cell polarization and function, as well as thymocyte development (39). CEBPD plays a pivotal role in controlling cellular anti-apoptotic processes, migration, reactive oxygen species generation, and cancer development, modulating these activities based on cell type and environmental conditions. It is particularly significant in autoimmune and inflammatory responses (40, 41).

While traditional CD8^+^ T cells are the primary effectors in tumor eradication, our findings indicate that Tc17 cells exhibit the weakest tumoricidal capacity among all CD8^+^ T cell subsets, surpassing only Treg cells. In contrast, the capacity of anti- inflammatory responses of Tc17 is relatively high (**Figure 2**). The aforementioned results imply that the functionality of Tc17 differs from that of the conventional effector CD8^+^ T cells, as they lack the ability to effectively kill tumor cells. Moreover, we deconvoluted the proportion of Tc17 in GC tumors from TCGA based on the scRNA-seq data as a reference using CIBERSORTx (42), and found that GC patients with a higher proportion of Tc17 exhibit poorer prognosis (**Figure 2**).

### Deciphering the trajectory of Tc17 differentiation

**Figure 3:**
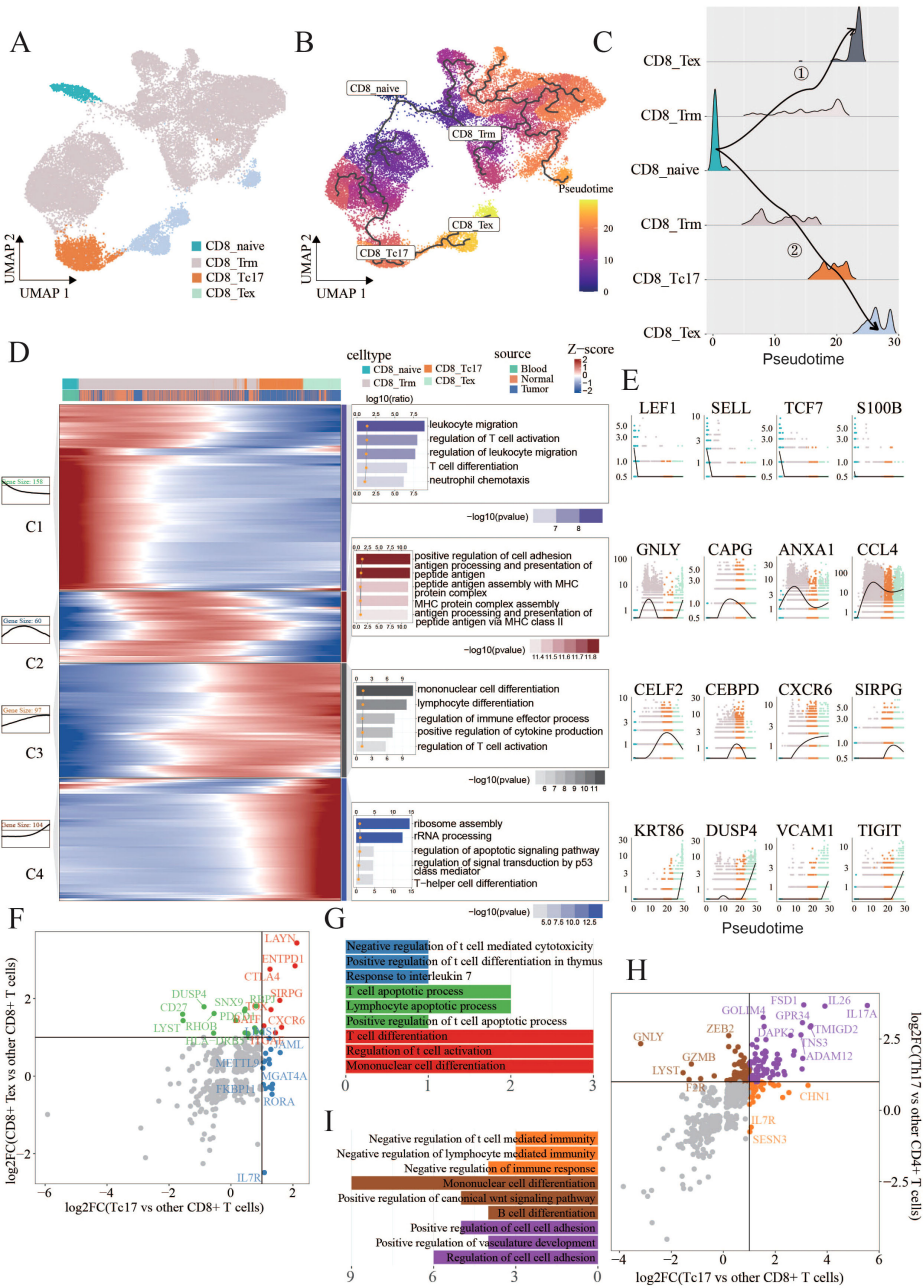
Tc17 cell differentiation trajectory **(A,B)** UMAP charts illustrate the distribution of four types of cells, labeled as CD8_Trm(22320), CD8_Tc17(2260), CD8_Tex(2310) and CD8_naive(743), as well as their pseudotime differentiation trajectories. **(C)** Ridge plots illustrate the density of cells across different differentiation trajectories. **(D)** Heatmaps, curve charts, and bar graphs delineate the expression profiles of genes fluctuating along pseudotime trajectories. The top differentially expressed genes (DEGs) are grouped into four clusters according to their distinctive expression signatures. The heatmap provides a visual representation of the expression levels of these prominent DEGs.Curve charts and bar graphs depict the expression patterns and the enrichment of associated Gene Ontology (GO) terms for each cluster. **(E)** Line charts illustrate the smoothed expression of certain genes corresponding to the modules on their left side. **(F,G)** Scatter plots display the intersection of differentially expressed genes (DEGs) specific to Tc17 cells when compared to other CD8^+^ T cells, and the DEGs specific to CD8^+^ exhausted cells when compared to other CD8^+^ T cells. Genes that are unique to CD8^+^ exhausted cells in contrast to Tc17 cells are colored green, those unique to Tc17 cells in contrast to CD8^+^ exhausted cells are colored blue, and genes common to both cell types are colored red. Bar charts indicate the pathways enriched by these three sets of genes **(H,I)** Scatter plots display the intersection of differentially expressed genes (DEGs) specific to Tc17 cells when compared to other CD8^+^ T cells, and the DEGs specific to Th17 cells when compared to other CD4^+^ T cells. Genes that are unique Th17 cells in contrast to Tc17 cells are colored brown, those unique to Tc17 cells in contrast to CD8^+^ exhausted cells are colored orange, and genes common to both cell types are colored purple. Bar charts indicate the pathways enriched by these three sets of genes.

To further investigate the reasons behind the diminished tumor-killing ability of Tc17 cells, we initially dissected their differentiation trajectory. Previous research has suggested that tissue-resident CD8^+^ T cells could transition into Tc17 cells in the TME (7). Therefore, we included CD8_Naive, CD8_Trm, CD8_Tc17, CD8_Tex in our study to observe the differentiation trajectories. The pseudotime analysis using Monocle3 proved that CD8_Naive T cells can progress towards exhaustion via two distinct trajectories: one directly transitions through the effector memory (Tem) phase to an exhausted state, while the other involves a Tc17 intermediate before reaching exhaustion. Notably, the pseudotime of Tc17 cells aligns with that of exhausted T cells, suggesting that Tc17 represents a terminal state prior to T cell exhaustion, potentially indicative of lost tumor-killing capabilities (**Figures 3A**).

By selectively isolating cells from the trajectory involved Tc17 cells, we aimed to elucidate the comprehensive gene repertoire governing their differentiation. Our analysis identified 419 dynamically expressed trend genes, categorized into four clusters based on their expression patterns, each with specific functional signatures (**Figures 3D**). Module 1 included genes associated with naive (LEF1,SELL) (43) and memory states (TCF7,NOSIP) (44, 45), enriched in the pathways related to T cell differentiation and migration. Module 2 displayed upregulation of cytotoxic mediators such as granzymes (GZMK, GZMB) (46), CCL4 and GNLY (47), alongside an enhancement in antigen presentation pathways. Module 3 harbored a repertoire of immune cell differentiation, including CELF2, CEBPD, CXCR6, and SIRPG (48–51). while Module 4 was characterized by exhaustion-associated genes such as KRT86, DUSP4, SNX9 and TIGIT (7, 52–54), and involved pathways linked to cellular depletion.

The aforementioned analysis revealed that Tc17 cells ultimately progress towards a state of exhaustion, losing their capacity to eliminate tumor cells. To ascertain the extent of exhaustion phenotype exhibited by Tc17 cells, we compared their gene expression signatures with those of exhausted CD8^+^ exhausted T cells. Eight genes that were commonly upregulated, including LAYN, CTLA4, CXCR6, ITGAE, SIRPG, ENTPD1, TOX and BATF, all associated with tumor progression or poorer prognosis (49, 51, 55–62) (**Figures 3F**, **Supplementary Table S3**). Notably, LAYN represents a valuable prognostic biomarker across various cancer types and also serves as an indicator of dysfunctional or exhausted T cells (58). CTLA4, immune checkpoint molecule, plays a crucial role in cell cycle regulation, modulation of T cell proliferation, and cytokine production. CXCR6 is implicated in T cells homing and immunosuppression within tumors (55). Further exploration revealed that high ITGAE and ENTPD1expression correlates with poor prognosis in renal cell and glioblastoma patients (56, 57). The comparative analysis of functional similarities and differences between Tc17 and conventional Th17 cells identified a series of genes specifically expressed in Tc17 cells that negatively regulate immune responses (**Figures 3H**, **Supplementary Table S3**). To further delineate the differences between Tc17 cells and cytotoxic T cells (IFNγ^+^ CD8^+^ T cells), we analyzed the IFNγ-high-expressing cell populations within our dataset. Utilizing a pseudo-bulk approach, we discovered that CD8_Tem exhibited significantly higher IFNγ secretion compared to other subpopulations. Based on this observation, our analysis revealed that the gene expression profiles of the cytotoxic T cell subset (CD8_Tem) and Tc17 cells were entirely distinct, with no commonly upregulated genes between them. Moreover, pathway analysis demonstrated markedly different immune response patterns: the cytotoxic T cell subset exhibited enhanced immune activation pathways, whereas the immune-related pathways observed in Tc17 cells were downregulated (**Supplementary Figure S3**). This reinforces the notion that Tc17 cells, despite being a CD8^+^ T cell subset, suppress immune responses rather than exerting anti-tumor effects (**Figure 2**). Previous research has shown that Tc17 cells can induce tumor cells to secrete CXCL12, thereby recruiting MDSCs that, in turn, exert an immunosuppressive effect on the cytotoxic activity of CD8^+^ T cells (20). The results of this study align with our findings that Tc17 cells suppress immune responses, thereby promoting tumor progression, however, the specific mechanisms through which Tc17 cells facilitate this process still warrant further exploration.

### Tc17 cells exhibit a pattern of coexistence with tumor cells

**Figure 4:**
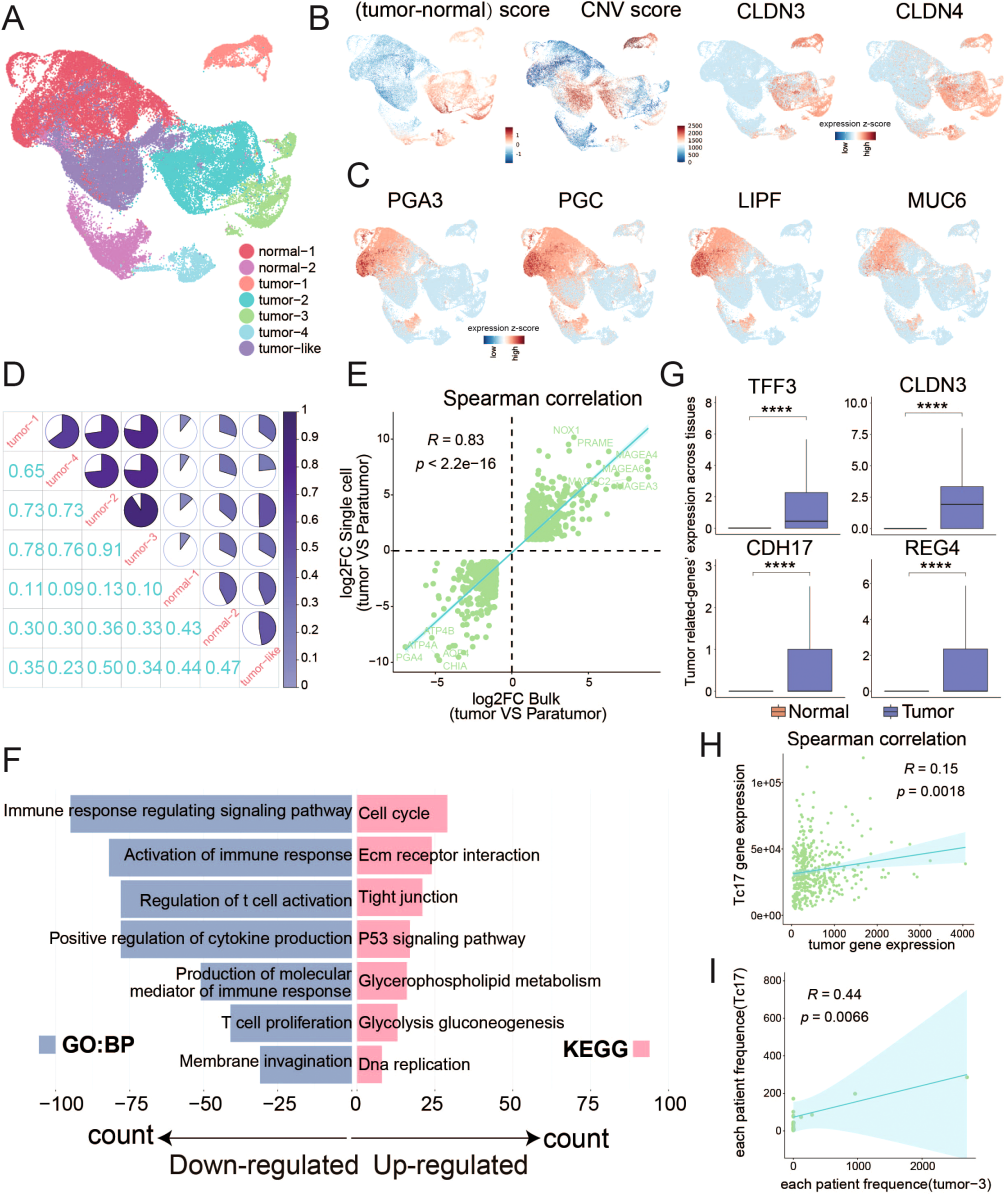
Distinguish malignant tumor cells from normal epithelial cells **(A)** UMAP plots display the clustering results of epithelial cells. **(B)** UMAP plots separately illustrate the results of tumor cell marker gene scoring minus the scoring of normal cell marker genes, the results of copy number variation (CNV), and the expression distribution of tumor cell markers (CLDN3 and CLDN4). **(C)** UMAP plots display the expression patterns of marker genes indicative of normal gastric function. **(D)** Pie chart illustrates the similarity among the expression profiles of the top 1000 highly variable genes within epithelial cell subpopulations. **(E)** Scatter plot demonstrates the correlation between the log2 fold change of differentially expressed genes from single-cell data (tumor cells vs normal cells) and TCGA data (tumor samples vs normal samples), with the p-value calculated using Spearman’s correlation. Differentially expressed genes were determined by corrected P-value < 0.01 and the absolute value of log2 foldchange >1. **(F)** Bar chart displays the GO or KEGG pathways enriched by the differentially expressed genes between tumor and normal cells in single-cell data. **(G)** Box plot illustrates the differential expression of genes between tumor and normal cells. **(H)** Scatter plot shows the correlation between the Tc17 marker genes and the expression of tumor cell marker genes. **(I)** Scatter plot shows the correlation between the proportions of Tc17 cells and tumor cell subgroup 3 in each sample. ****P < 0.0001.

In light of the aforementioned analysis revealing that the presence of Tc17 cells in TME is a risk factor for the survival of GC patients (**Figure 2**), and considering the distinct anti-inflammatory characteristics of Tc17 cells compared to conventional CD8^+^ T cells, we further explored their role in tumorigenesis. Since GC malignant cells originate from epithelial cells, we isolated epithelial cells for tumor cell identification. By assigning a tumor markers score (9) in GC and assessing copy number variations using InferCNV for each cell, we identified four subclusters of tumor cells with both highest tumor scores and significant copy number alterations, two normal clusters, and one tumor-like cluster (**Figure 4A**, **Supplementary Figures S3B, C**, **Supplementary Tables S4**, **S5**). Consistent with expectations, normal clusters retained the characteristic functional attributes of gastric tissue, exhibiting distinct expression patterns of genes associated with gastric physiology, including pepsinogens (PGA3, PGC), the lipase gene (LIPF), and several mucin genes (MUC6) (63) (**Figure 4**). Correlation analysis also revealed significant disparities in gene expression profiles between tumor and normal cells (**Figure 4**). To validate the tumor cell clusters identified via scRNA-seq, we examined if similar gene expression signatures were present in bulk RNA-seq data from GC samples in TCGA. We identified differentially expressed genes (DEGs) from scRNA-seq by comparing the tumor clusters with normal clusters and performed a parallel analysis using bulk RNA-seq data by comparing tumor samples with adjacent non-tumor samples. There was a high correlation between the expression of tumor-driven DEGs identified in the scRNA-seq data and bulk RNA-seq datasets, indicating that the gene expression patterns of the tumor cell clusters closely mirrored those of the tumor tissue (**Figure 4**).

Functional enrichment analysis of DEGs between tumor cells and normal cells revealed a significant up-regulation of pathways related to the cell cycle and cell adhesion. Conversely, pathways associated with immune response were found to be downregulated, pointing to a diminished immune response within TME (**Figure 4**, **Supplementary Figure S3D**). Notably, genes such as TFF3, CLDN3, CDH17, and REG4 exhibited specific expression in tumor cells, linking to the cancer progression (**Figure 4**). Trefoil factor 3 (TFF3) modulates cancer development through pathways like MAPK/ERK and PI3K/AKT (64). Claudin-3 (CLDN3) is associated with colorectal cancer progression (65), while Cadherin-17 (CDH17) correlates with unfavorable prognosis in various cancers (66). Regenerating islet-derived type 4 (REG4) enhances invasiveness and migration of gastric tumor cells (67). Additionally, a significant correlation between the expression of tumor cell signature genes and Tc17 marker genes was observed (**Figure 4**). Our findings also indicate that Tc17 cells are likely to coexist with tumor subtype 3 (**Figure 4**, **Supplementary Figure S2C**), highlighting a state of coexistence that influences cancer progression.

### The CXCL16-CXCR6 axis in tumor cells and Tc17 cells: a potential driver of gastric cancer progression

**Figure 5:**
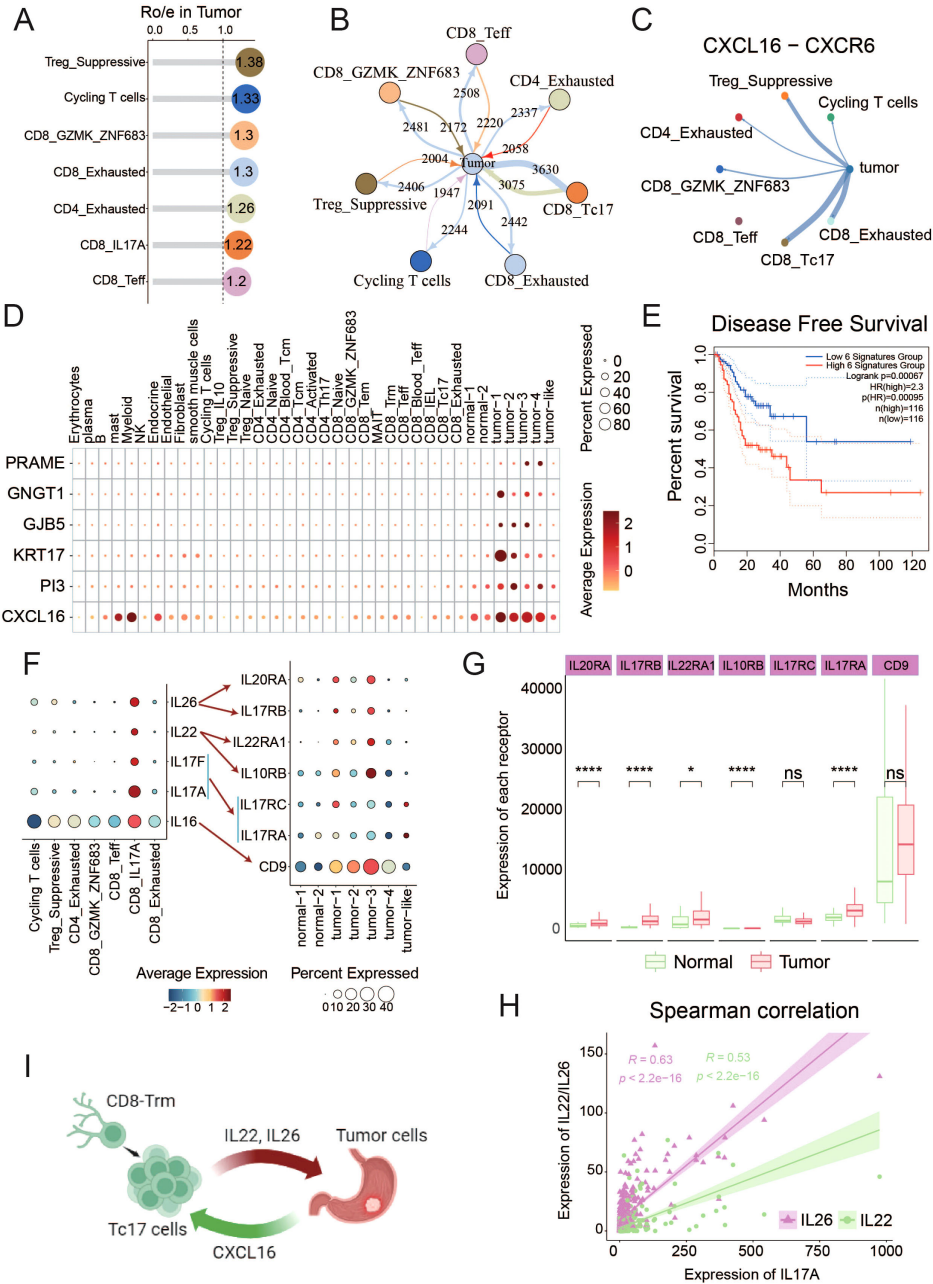
Interaction between tumor cells and Tc17 cells **(A)** The Ro/e values in the lollipop chart reflect the enrichment levels of T cell subsets within the TME. **(B)** The iTALK predictions illustrate the number of interactions between different cell types and tumor cells. Thicker lines represent a higher number of interactions. **(C)** CXCL16-CXCR6 interaction Intensity of interaction between tumor cells and T cell subtypes in TME. **(D)** Dot plot reflects the expression of CXCL16 and tumor cells marker genes in all tumor-enriched cell types. **(E)** The Kaplan-Meier curve describes disease-free survival (DFS) of GC patients by grouping the overall expression of six genes in **Figure 5D**. Red indicates patients with high expression levels and green indicates patients with low expression levels. The p-value is calculated using the Log-rank test. **(F)** The expression of interleukins IL16, IL17A, IL17F, IL22, and IL26 in T cells, as well as the expression of their corresponding receptors in epithelial cell subpopulations. **(G)** The expression differences of interleukins corresponding receptors between tumor and normal samples in TCGA data. **(H)** The correlation between the expression of IL22/IL26 and IL17A in tumors in TCGA data. **(I)** Schematic diagram of the interaction between Tc17 cells and tumor cells. ns, not significant. *P < 0.05, ****P < 0.0001.

To explore the intercellular interactions between tumor and T cells within TME, we applied Ro/e scoring to identify T cell subclusters enriched in the tumor tissues, specifically focusing on CD4_Exhausted, CD8_Teff, CD8_Tc17, Treg_Suppressive, Cycling T cells, CD8_GZMK_ZNF683 and CD8_Exhausted (**Figure 5**, **Supplementary Figure S2D**). It is reasonable to assume that the majority of T cells enriched in TME exhibited an exhausted and immunosuppressive state. In this study, we focus on Tc17 cells, which also show a reduced capacity for tumor-killing and a coexistence with tumor cells (**Figures 4H**). To ascertain the strength of the connection between tumor cells and these tumor-enriched T cells, we employed iTALK software to establish a cellular communication network based on potential ligand- receptor interactions, revealing that Tc17 cells harbored the highest number of interactions with tumor cells (**Figure 5**). Further analysis identified strong interactions between Tc17 cells and tumor cells via CXCL pathway (**Supplementary Figure S4**). Upon further analysis of the potential gene pairs that could impact this pathway, we identified that the CXCL16-CXCR6 axis is more intense in tumor cells and immune-suppressive T cells (CD8_Exhausted, Treg_Suppressive and Tc17) compared to other T cell subclusters, with the highest interaction strength observed with Tc17 cells (**Figure 5**, **Supplementary Figure S4B**). In the context of glioblastoma, the CXCL16-CXCR6 axis mediates interactions between T cells and tumor-associated macrophages, impairing T-cell functionality and enhancing immunosuppression (55). In breast cancer, this axis promotes migration and invasion of cancer cells via the activation of Src, FAK, and ERK1/2 signaling pathways (68). Survival analysis, which incorporated scoring for CXCL16 and five signature genes specifically overexpressed in tumor cells, discovered a significant association between high CXCL16 expression and poor prognosis in GC patients (**Figures 5D**).

Interleukins constitute a pivotal class of cytokines within the immune system, acting as critical messengers for cellular communications within TME (69). We observed that IL-16, IL-17, IL-22, and IL-26 are specifically expressed in Tc17 cells, and their corresponding receptors are also highly expressed in tumor cells (**Figure 5**, **Supplementary Figure S4D**). IL-16 is involved in the proliferation and transformation of malignant cells (70). IL-17 stimulates the proliferation and self-renewal of ovarian cancer stem cells through the NF-κB and MAPK pathways. The expression and activation of IL-17RA can directly promote the tumorigenesis of colon cancer cells (71). IL-22 enhances migration and maintains stemness in cancer cells across multiple cancer types (69), and IL-26 has been linked to EGFR-TKI resistance in triple-negative breast cancer (72). Subsequent analysis revealed a robust positive correlation among the expressions of IL-22, IL-26, and IL-17A, with their corresponding receptors were notably elevated in the tumor samples (**Figure 5G**, **Supplementary Figure S4C**). These results demonstrate that a series of interleukins (IL-16, IL-17, IL-22, and IL-26) secreted by Tc17 cells could collaboratively act on tumor cells, promoting tumor occurrence and progression.

In conclusion, our findings suggest that tumor cells can orchestrate the recruitment of Tc17 cells via the CXCL16-CXCR6 axis in GC. Once in close proximity to the tumor cells, these Tc17 cells engage in a reciprocal signaling cascade, secreting interleukins (IL-16, IL17, IL-22, and IL-26), thereby forming a vicious cycle that promotes the progression of tumor cells (**Figure 5**).

### Spatial transcriptomic data confirms the proximity of tumor cells and Tc17 cells

**Figure 6:**
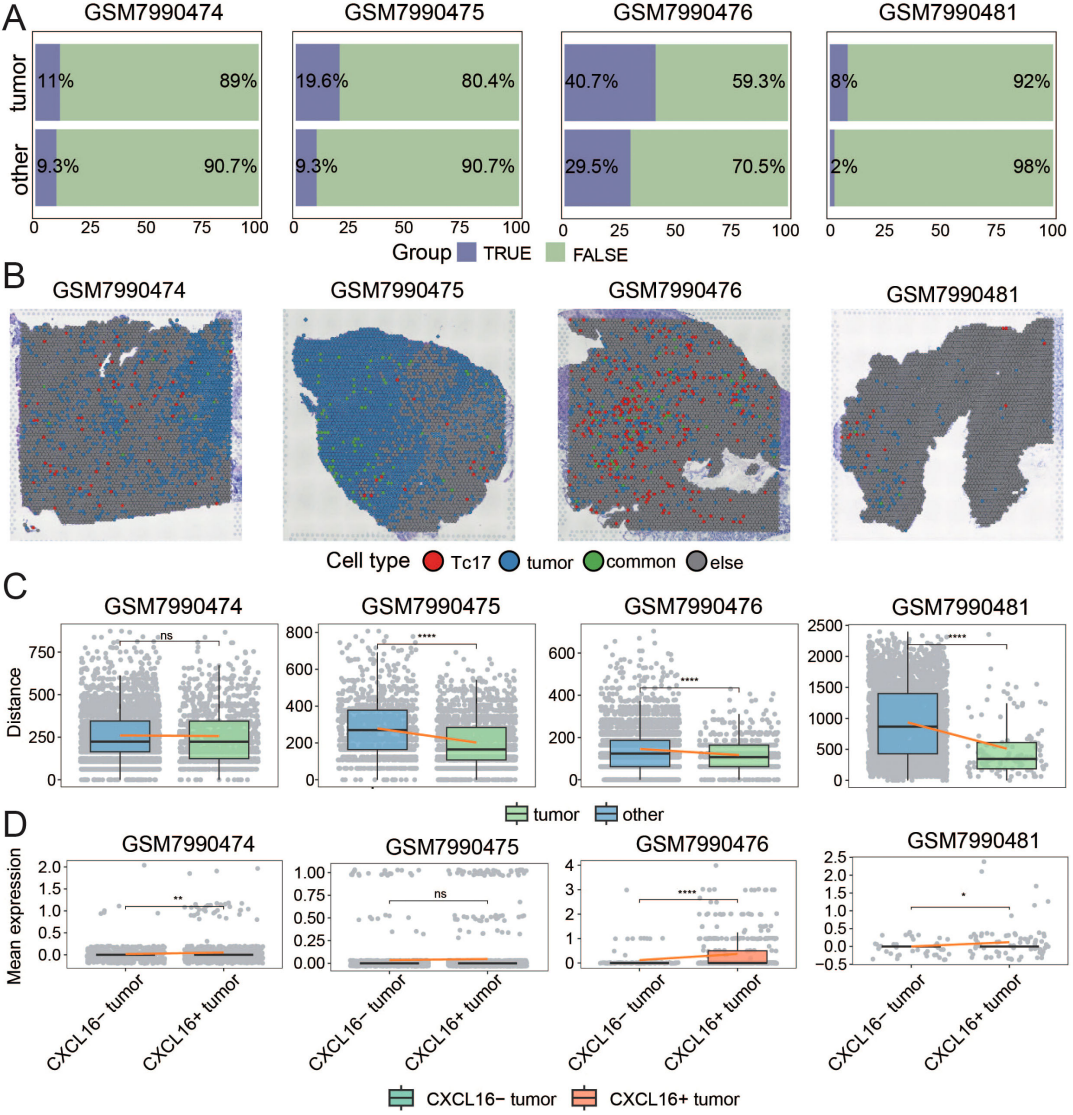
Spatial Transcriptomic Data Confirms the Proximity of Tumor Cells and Tc17 Cells **(A)** The bar chart illustrates the proportion of Tc17 cells surrounding tumor regions (In the same spot) and non-tumor regions. **(B)** Histologic image of the case is shown with the spatial map illustrating the distribution of Tc17 cells, tumor cells, and spots co-localized with both Tc17 cells and tumor cells. **(C)** The boxplot shows the distance between tumor cells and the nearest Tc17 cells, as well as the distance between non-tumor cells and the nearest Tc17 cells. **(D)** The boxplot illustrates the average expression of CXCR6 in Tc17 cells surrounding CXCL16^+^ tumor cells and CXCL16^−^ cells. ns, not significant. *P < 0.05, **P < 0.01, ****P < 0.0001.

To further validate our findings, we analyzed ten spatial transcriptomics samples (**Supplementary Table S1**). We first identified Tc17 cell localization by colocalizing T cell markers (CD8A/CD8B), CD8^+^ T cell markers (CD8A/CD8B), and Tc17 specific markers (IL17A/RORC). Tumor cell regions were demarcated using gastric cancer-specific marker genes (KRT17/PRAME/GNGT1/GJB5/PI3). Strikingly, Tc17 cells were detected in nine of the ten samples. Quantification revealed that in seven samples, the density of Tc17 cells surrounding tumor cells (within the same spatial spot) was significantly higher than in distal regions (**Figure 6**, **Supplementary Figure S5A**).

For a conservative and rigorous analysis, we focused on four samples in which the proportion of Tc17 cells in the tumor-adjacent regions exceeded 5% (**Figure 6**). Histological examination confirmed spatial coexistence of Tc17 cells and tumor cells in all four cases (**Figure 6**, **Supplementary Figure S5B**). To further analyze their spatial relationship, we calculated the Euclidean distance between each tumor cell and its nearest Tc17 cell, as well as the distance between non-tumor cells and their nearest Tc17 cells. The results showed that in three samples, tumor cells were significantly closer to Tc17 cells (**Figure 6**, **Supplementary Figure S5C**). Notably, three of these four samples were EBV-positive, suggesting a potential association between EBV infection and Tc17 cell enrichment.

To validate the hypothesis that tumor cells recruit Tc17 cells via the CXCL16-CXCR6 axis, we categorized spots with tumor cells into CXCL16^+^ and CXCL16^-^ groups and compared the expression of CXCR6 in surrounding Tc17 cells. The results indicated that Tc17 cells adjacent to CXCL16^+^ tumor cells exhibited higher CXCR6 expression (**Figure 6**, **Supplementary Figure S5D**). These findings collectively confirm the functional role of the CXCL16-CXCR6 axis in the recruitment of Tc17 cells by tumor cells.

### *In Vitro* validation of the critical roles of IL-17A and IL-26 in the gastric cancer microenvironment

**Figure 7:**
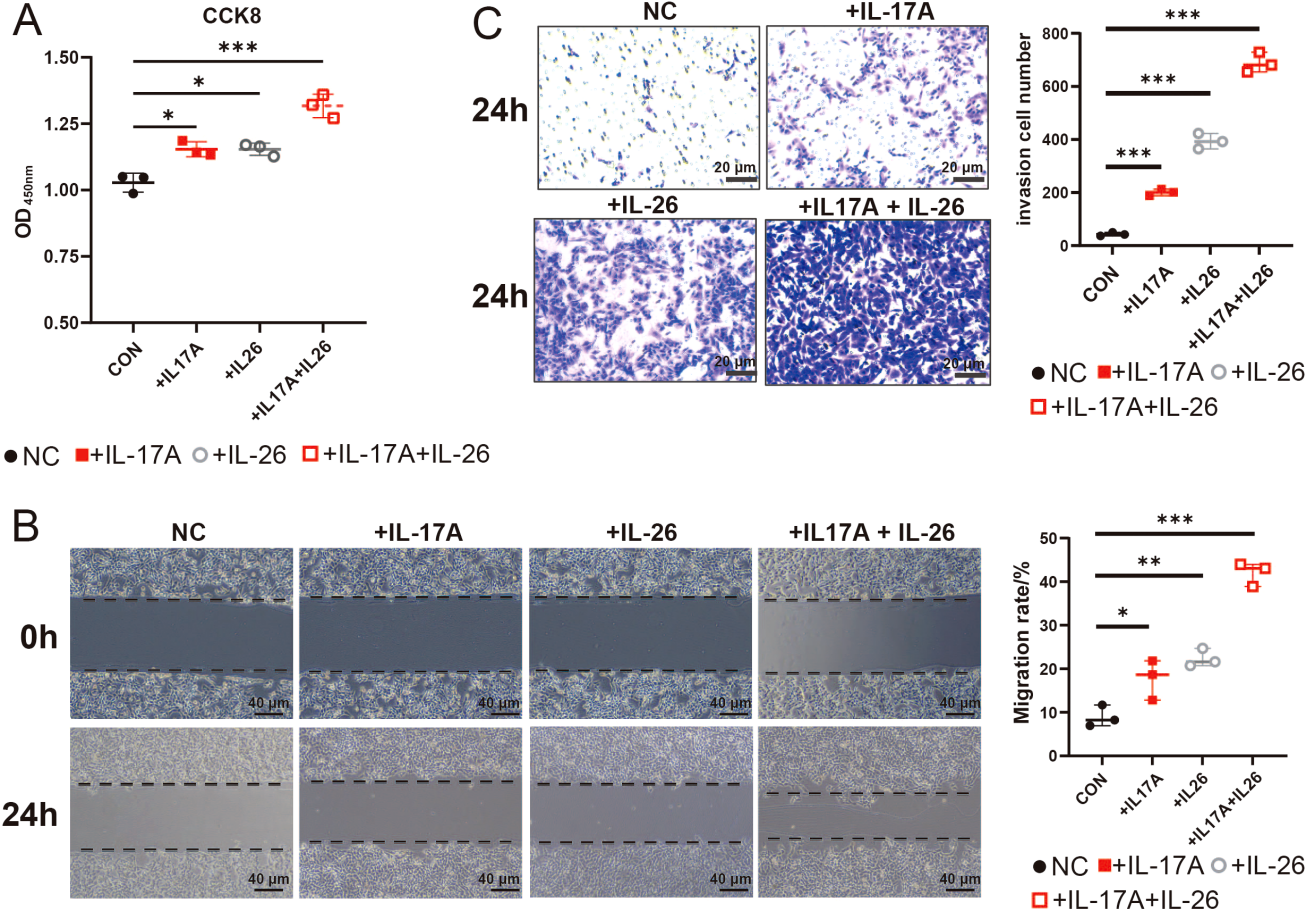
CCK-8,Wound Healing Assays and Transwell Assays to Evaluate the Effects of IL17A and IL26 on Tumor Cells **(A)** The effects of IL17A (50 μg/mL), IL26 (50 μg/mL) and their combined treatment on the proliferation capacity of gastric cancer cells were evaluated by CCK-8 cell proliferation assay. **(B)** The effects of IL17A (50 μg/mL), IL26 (50 μg/mL) and combined treatment on the migration ability of gastric cancer cells were analyzed by scratch test. Cells were photographed at 0 h and 24 h after scratches, and the percentage of migration area was calculated. **(C)** Transwell invasion assay showing the effects of IL-17A (50 μg/mL), IL-26 (50 μg/mL), and their combination on gastric cancer cell invasion after 24 h. *P < 0.05, **P < 0.01, ***P < 0.001.

Based on transcriptome data analysis revealing elevated expression of IL17A and IL26 in gastric cancer tissues (**Figure 5**), we systematically evaluated the impact of these two cytokines on malignant biological behaviors of GC tumor cells through *in vitro* functional experiments. Stimulation of GC cells with either IL17A or IL26 alone resulted in a notable enhancement of cell proliferation compared to negative control group (NC). Intriguingly, co-stimulation with both cytokines led to an even more pronounced proliferative effect, suggesting a synergistic interaction (**Figure 7**). Similarly, wound healing assays demonstrated that IL17A and IL26 individually promoted the migratory capacity of tumor cells, while combined treatment elicited the highest migration rate (**Figure 7**). Furthermore, transwell invasion assays confirmed the pro-invasive roles of IL17A and IL26. Cells exposed to both cytokines exhibited significantly greater invasive capabilities than those treated with either cytokine alone (**Figure 7**). Collectively, these findings reveal that IL17A and IL26 not only individually enhance malignant phenotypes of GC cells but also act synergistically to potentiate tumor progression.

## Discussion

Gastric cancer (GC) presents significant treatment challenges due to its relatively low immunogenicity and limited response to immunotherapy. Unlike other epithelial cancers with high mutation rates, GC has historically been considered less responsive to immune-based treatments, restricting the benefits of immune checkpoint blockade (ICB) to only a small subset of patients (4, 73). Although combining immunotherapy with chemotherapy has been explored in various clinical settings to improve outcomes, determining the optimal chemotherapy regimen remains a complex task, further complicated by potential adverse effects. A deeper understanding of the gastric cancer immune microenvironment is crucial for refining therapeutic strategies, identifying biomarkers for patient stratification, and developing more effective combination treatments. Further research is essential to overcome these challenges and fully harness the potential of immunotherapy in GC.

Recent studies have highlighted the critical role of Tc17 cells in shaping the immunosuppressive TME across various cancers. Zhuang et al. demonstrated that in gastric cancer, Tc17 cells promote the secretion of CXCL12 by tumor cells via IL-17, subsequently recruiting MDSCs through the CXCL12-CXCR4 axis (20). Similarly, Lee et al. reported that in hepatocellular carcinoma, IFNγ^−^ Tc17 cells recruit Tregs via CCL20 overexpression, while also facilitating angiogenesis through IL-17A and mediating immunosuppression via CTLA-4/PD-1 and IL-10R pathways, collectively driving tumor progression and poor prognosis (74). In pancreatic ductal adenocarcinoma, Picard et al. revealed that Tc17-derived IL-17A and TNF activate cancer-associated fibroblasts (CAFs), inducing their differentiation into inflammatory CAFs (iCAFs), thereby establishing a Tc17-iCAF feedback loop that exacerbates immunosuppression and tumor progression (75). Our study not only expands the understanding of Tc17 cells in contributing to the immunosuppressive microenvironment but also proposes novel mechanisms by which they act within the TME. Unlike previous studies, we suggest that GC tumor cells can directly interact with Tc17 cells. Initially, tumor cells secrete CXCL16 to recruit Tc17 cells, thereby enhancing their accumulation within the TME. Subsequently, Tc17 cells secrete IL-17A and IL-26, which further promote tumor progression and establish a vicious cycle, collectively constructing the immunosuppressive microenvironment in GC

CXCL16 plays a pivotal role in a variety of cancers, such as GC, glioblastoma multiforme, lung cancer, lymphoma, nasopharyngeal carcinoma, hepatocellular carcinoma, pancreatic cancer, and breast cancer, among others. Its role is dualistic: it can facilitate the migration and invasion of cancer cells and is instrumental in increasing tumor-infiltrating lymphocytes, regulating angiogenesis, controlling cellular behavior, and guiding the migration of tumor cells to target sites. However, in certain cancer types like renal cell carcinoma and breast cancer, CXCL16 may also reduce cancer cell proliferation (76, 77). Reports have indicated that CXCR6 expression is elevated in some T cells, and these CXCR6^+^ T cells are recruited into tumor tissues through a CXCL16 gradient. In our current study, we have demonstrated through various approaches that GC tumor cells highly express CXCL16, while Tc17 cells, a subset of T cells, highly express CXCR6. This suggests that tumor cells may recruit Tc17 cells to the TME via the CXCL16-CXCR6 axis. Additionally, Tc17 cells stimulate the release of the chemokine CXCL12 from tumor cells, which in turn recruits CXCR4^+^ MDSCs. The accumulation of MDSCs inhibits the activation of tumor-infiltrating cytotoxic T lymphocytes (CTLs), thereby supporting the establishment of an immunosuppressive TME by Tc17 cells (37). Therefore, targeting CXCL16 may be a potential target for the treatment of GC, but the specificity of different tissues should be considered when designing the target.

IL-22 exerts a complex role in epithelial malignancies such as lung, liver, pancreatic, colorectal, and GC, acting as a promoter of tumorigenesis. It stimulates cell migration, the expression of matrix metalloproteinases (MMPs), angiogenesis, and the development of dysplastic tissues. Interestingly, in the context of inflammation-driven cancer models, IL-22 may decelerate tumor growth, but paradoxically, once a tumor is established, it can hasten the progression of cancer (78). Furthermore, IL-26 is known to induce cells that produce IL-22, potentially reducing the cytotoxic T cell function through the activation of anti-apoptotic proteins, thus fostering tumor growth (79). Our data indicate that Tc17 cells highly express a range of cytokines, notably IL-16, IL-17A, IL-17F, IL-22, and IL-26, and it is these five cytokines for which receptors are also highly expressed in tumor cells. Notably, IL-22 and IL-26 are co-expressed with IL-17A, and IL-16 shows a trend towards co-expression with IL-17A. Similarly, this finding has been confirmed in the TCGA dataset. Subsequently, we demonstrated through CCK-8 assays, scratch wound healing assays, and Transwell invasion assays that both IL-17A and IL-26 significantly enhanced the proliferation, migration, and invasive capacities of gastric cancer cells, thereby facilitating gastric cancer progression. These findings collectively suggest that Tc17 cell-derived IL-17A and IL-26 may exert profound regulatory effects on tumor cell malignancy.

Here, we further recognize that γδ T cells and group 3 innate lymphoid cells (ILC3s) serve as innate sources of IL-17, playing crucial roles in mucosal defense against pathogens while also contributing to pathological inflammation and autoimmune disorders (80, 81). Specifically, we now acknowledge that IL-17 can be secreted by a variety of immune cell types, including Th17 cells, γδ T cells, and ILC3s. While these populations share the ability to produce IL-17, they may exert distinct functional roles within the tumor microenvironment. Future investigations employing broader profiling technologies or specialized enrichment strategies to capture all IL-17-secreting cells would be highly valuable for dissecting the functional heterogeneity of these cell types and advancing our understanding of immune regulation in both cancer and inflammatory diseases.

Although integrated analysis of single-cell RNA sequencing and spatial transcriptomics data suggested a potential interaction between tumor cells and Tc17 cells via the CXCL16-CXCR6 axis, it is critical to acknowledge that these conclusions remain computationally inferred and lack experimental validation. Notably, our spatial transcriptomic datasets were deficient in detecting IL16, IL17, IL22, and IL26 expression—a limitation likely stemming from the inherent technical constraints of current spatial profiling platforms in reliably capturing secreted factors. This gap precluded spatial validation of the proposed molecular interactions. Future studies will experimentally validate these molecular interactions to identify robust therapeutic targets and advance our understanding of TME dynamics. Our study elucidates the immunosuppressive role of Tc17 cells in the gastric cancer microenvironment, particularly their recruitment by tumor cells via the CXCL16-CXCR6 axis and subsequent promotion of tumor progression through the secretion of cytokines such as IL-17A and IL-26. These findings pave the way for the development of novel therapeutic strategies. First, targeting the CXCL16-CXCR6 axis may be an effective therapeutic approach, as inhibiting this axis could reduce the recruitment and accumulation of Tc17 cells in the tumor microenvironment, thereby mitigating their immunosuppressive effects. Future research could focus on developing inhibitors against CXCL16 or CXCR6 and evaluating their efficacy in gastric cancer treatment. Second, antibodies or small molecule inhibitors targeting IL-17A and IL-26 secreted by Tc17 cells may effectively block their tumor-promoting actions. When combined with existing immune checkpoint inhibitors, such as anti-PD-1/PD-L1 therapies, these strategies hold promise for improving the response rates of gastric cancer patients to immunotherapy. Additionally, our findings suggest that the presence of Tc17 cells is associated with poor prognosis in gastric cancer patients, indicating that Tc17 cells and their secreted cytokines could serve as biomarkers for prognostic assessment and prediction of treatment response in gastric cancer. In summary, our study not only enhances the understanding of Tc17 cells’ role in gastric cancer but also provides a theoretical foundation for the development of new therapeutic strategies and biomarkers. Future research will be dedicated to translating these fundamental discoveries into clinical applications to improve treatment outcomes and prognosis for gastric cancer patients.

## Data Availability

The original contributions presented in the study are included in the article/**Supplementary Material**. Further inquiries can be directed to the corresponding author/s.
