# Supplementary figures and images for: A novel Tc17 population recruited by tumor cells promotes tumor progression in gastric cancer
Source: Front Oncol. 2025 May 16;15:1592328. doi: 10.3389/fonc.2025.1592328 (PMC12122342; doi:10.3389/fonc.2025.1592328)

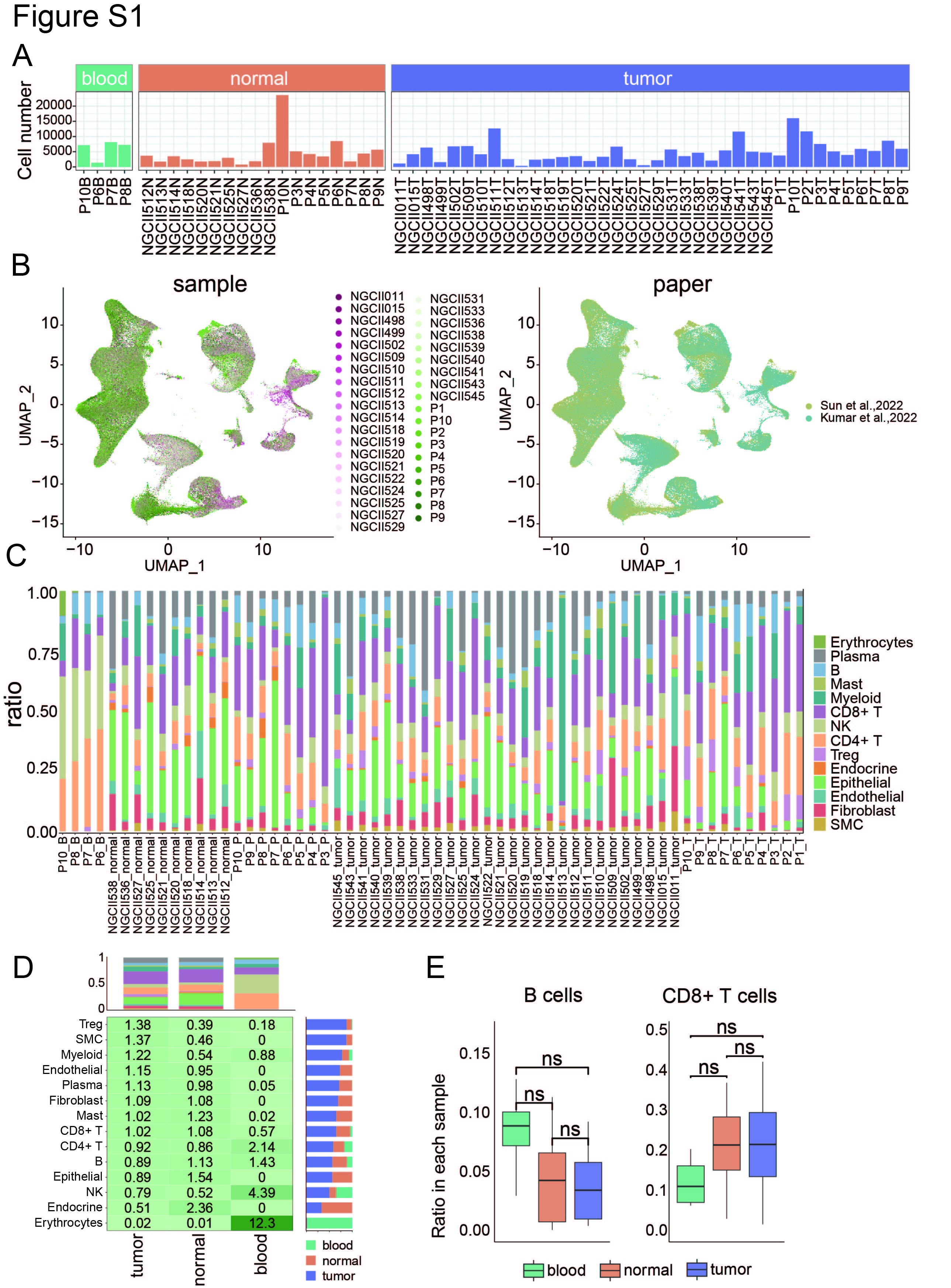

Supplement: Supplementary file 1 [file Image1.jpeg]

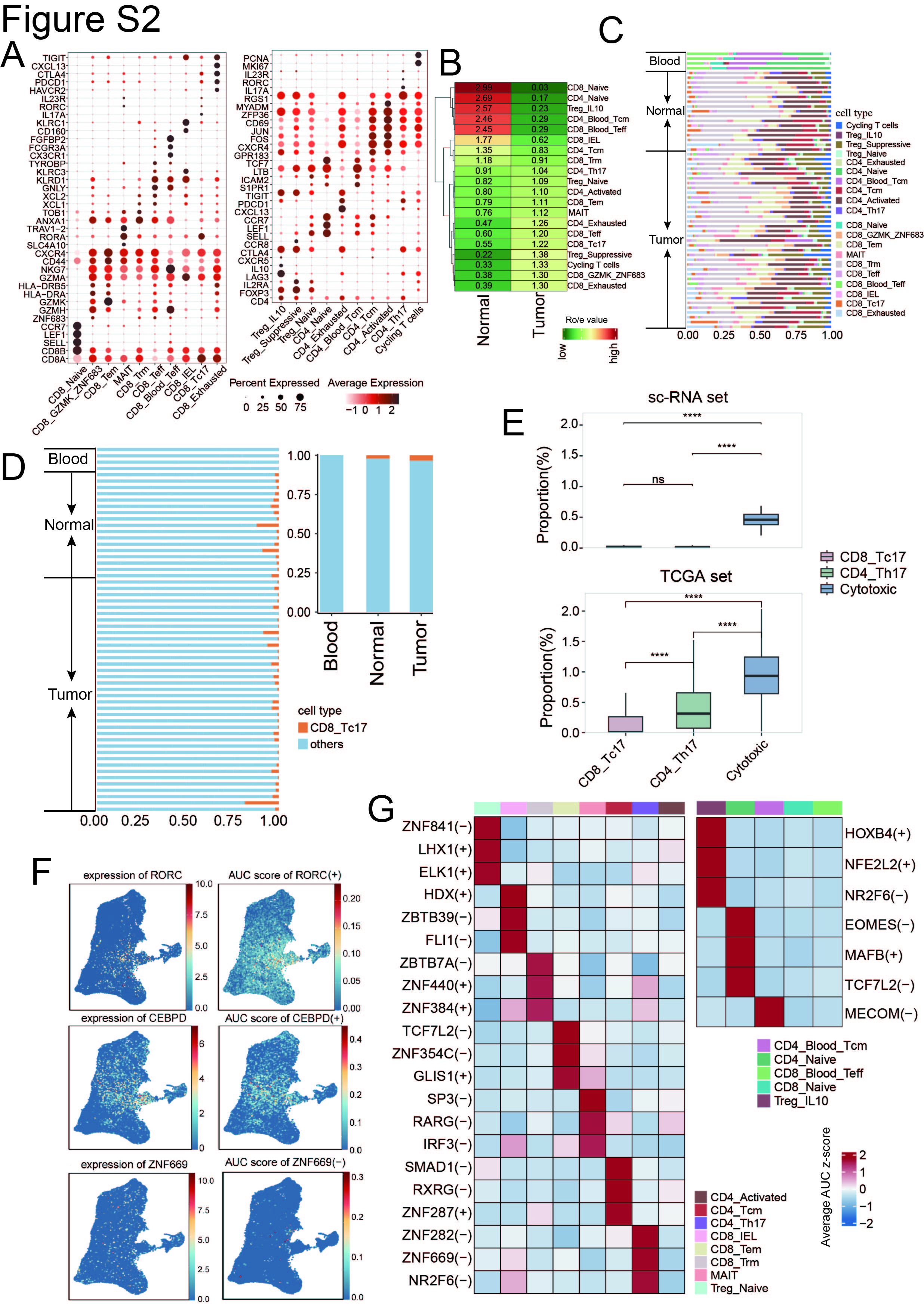

Supplement: Supplementary file 2 [file Image2.jpeg]

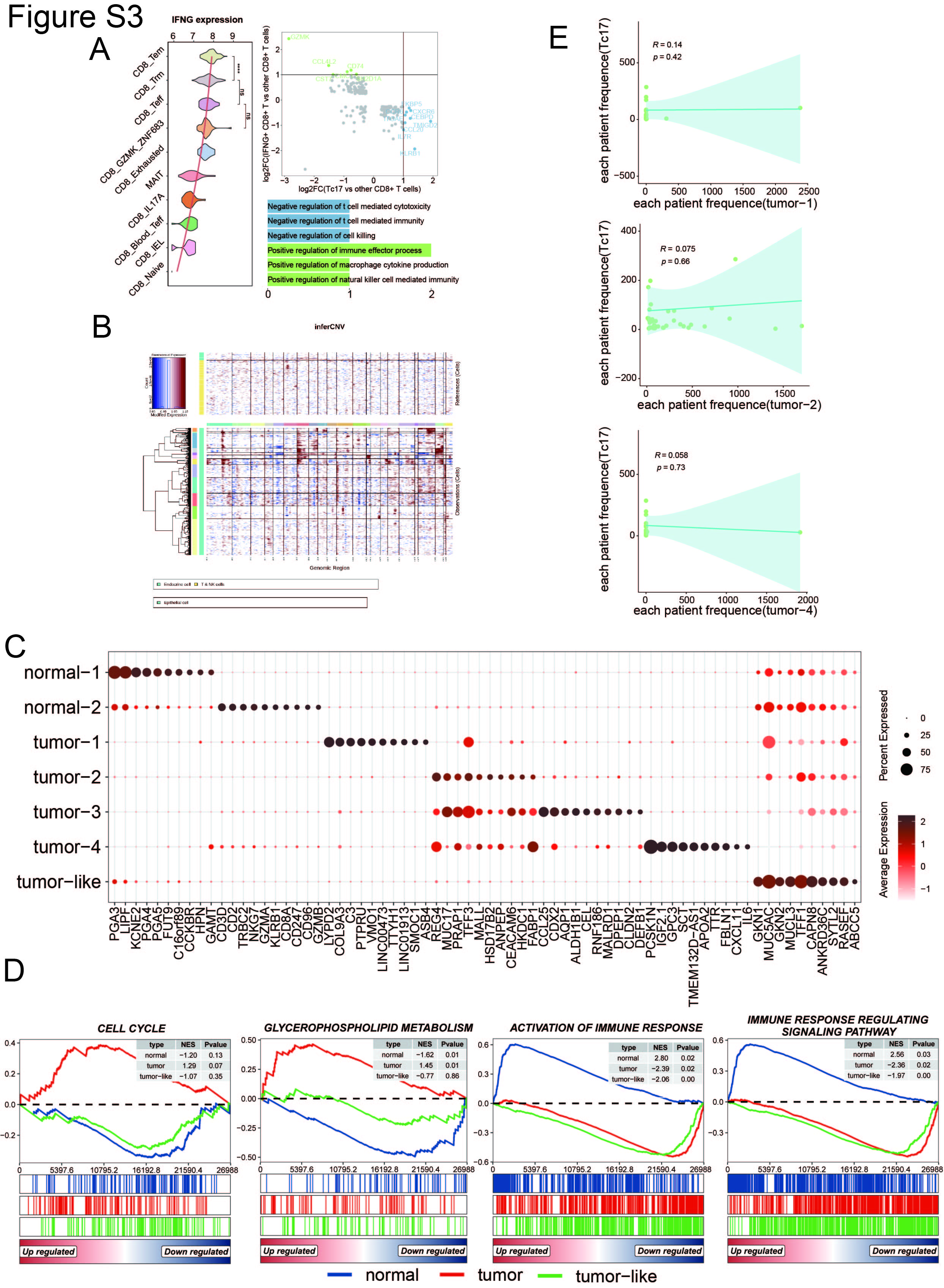

Supplement: Supplementary file 3 [file Image3.jpeg]

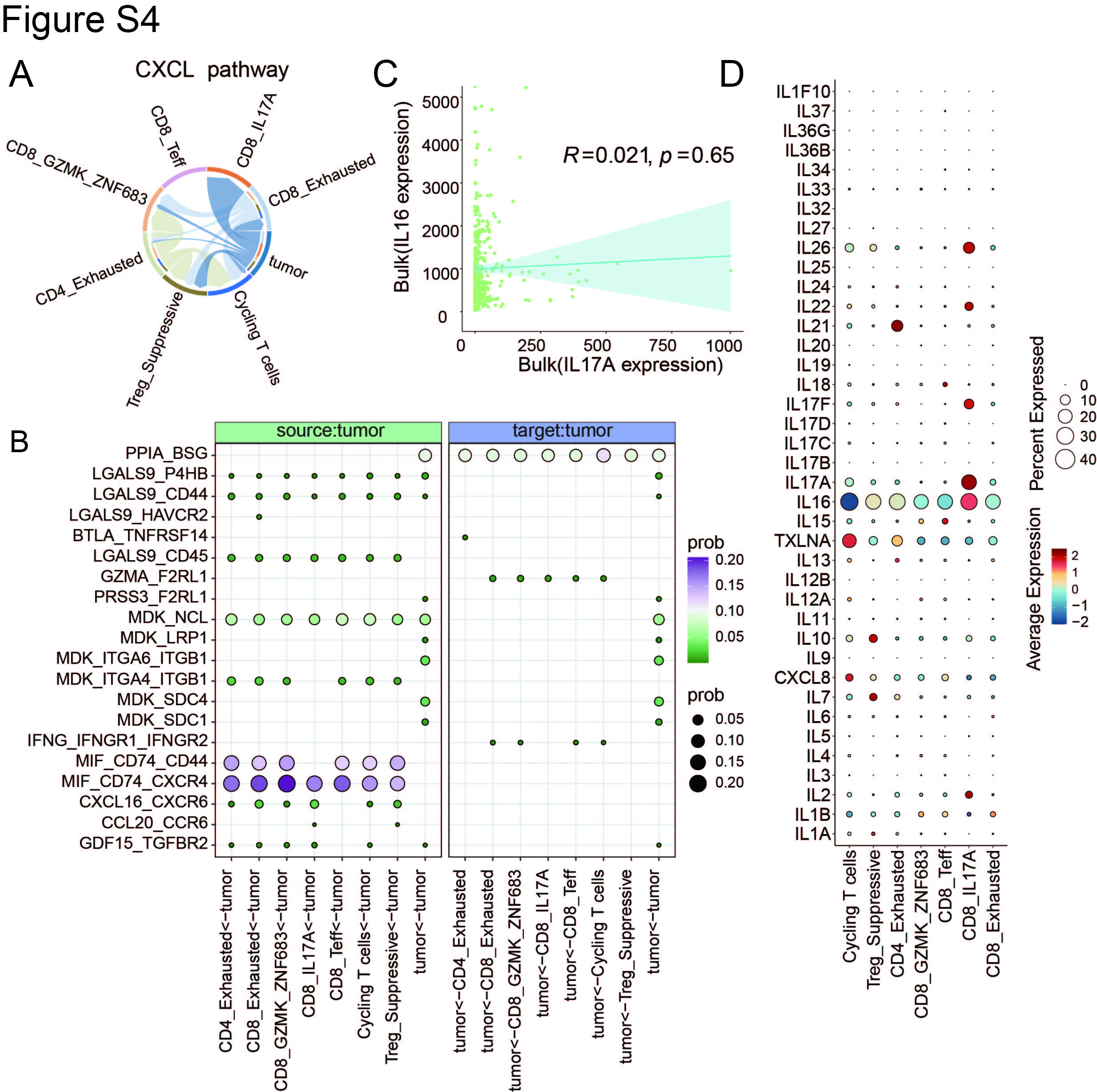

Supplement: Supplementary file 4 [file Image4.jpeg]

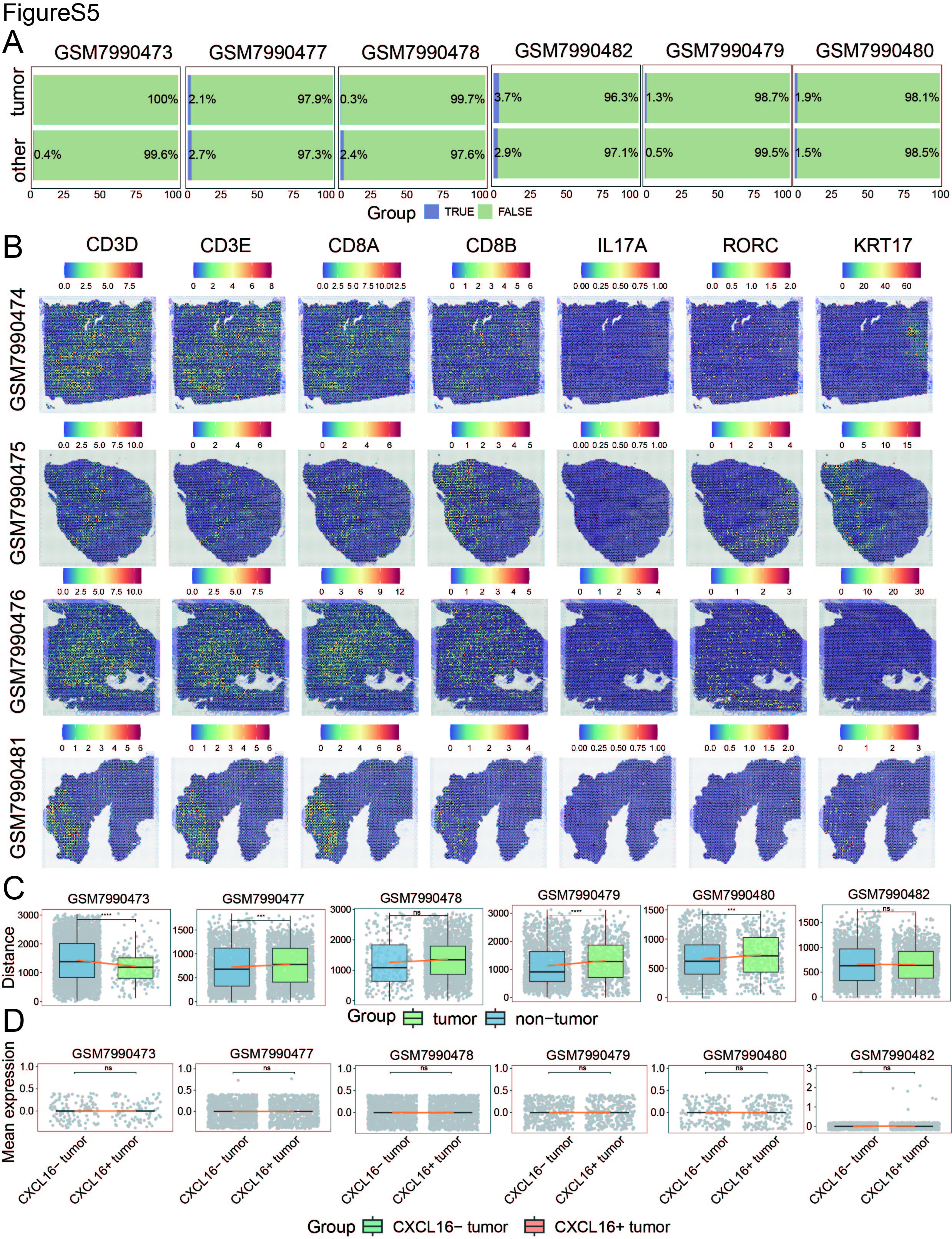

Supplement: Supplementary file 5 [file Image5.jpeg]
