# Supplementary material for: A novel Tc17 population recruited by tumor cells promotes tumor progression in gastric cancer
Source: Front Oncol. 2025 May 16;15:1592328. doi: 10.3389/fonc.2025.1592328 (PMC12122342; doi:10.3389/fonc.2025.1592328)
Supplement: Supplementary file 11 [file Table6.docx]

Table S6: List of abbreviations

| Abbreviation  TME  GC  PD-1  CAR-T  scRNA-seq  ICB  MDSCs  CCI  Tem  Trm  MAIT  IEL  Tcm  Treg  CTL  Tfh  Tfr  IL  CXCL16  CXCR6  DEGs  CNV  Ro/e | Full term  Tumor microenvironment  Gastric cancer  Programmed Cell Death Protein 1  Chimeric Antigen Receptor T-cell Therapy  Single cell RNA sequencing  Immune Checkpoint Blockade  Myeleloidderived suppressor cells  Cell-Cell interaction  Effector Memory T cells  Tissue-Resident Memory T cells  Mucosal-Associated Invariant T cells  Intraepithelial Lymphocytes  Central Memory T cells  Regulatory T cells  Cytotoxic T Lymphocytes  T Follicular Helper Cells  T Follicular Regulatory Cells  Interleukin  C-X-C Motif Chemokine Ligand 16  Motif Chemokine Receptor 6  Differentially Expressed Genes  Copy Number Variation  the ratio of observed over expected cell numbers |
| --- | --- |
