# Supplementary material for: A novel Tc17 population recruited by tumor cells promotes tumor progression in gastric cancer
Source: Front Oncol. 2025 May 16;15:1592328. doi: 10.3389/fonc.2025.1592328 (PMC12122342; doi:10.3389/fonc.2025.1592328)
Supplement: Supplementary file 12 [file Table7.docx]

**Extended Figure Legends**

**Figure S1. Dynamic alterations in cell populations within the tumor microenvironment**

(A) The bar chart shows the number of cells from different samples.

(B) The UMAP illustrates the dimensionality reduction clustering results for different samples (left) or data sources (right) after the batch effect is removed.

(C) The bar chart shows the proportion of each cell type in each sample.

(D) The central figures indicate the distribution of various big cell-types according to Ro/e scores. The bar chart at the top shows the proportion of individual cell types from different tissue sources, and the bar chart on the right illustrates the percentage of different tissue sources within each cell type.

(E) The box plot shows statistically significant differences in the proportion of CD8+ T cells and B cells in each sample between different tissue sources. P-values were calculated by Wilcoxon rank-sum test. *P < 0.05, **P < 0.01, ***P < 0.001, ****P < 0.0001.

**Figure S2. The profiling of T cell subsets**

1. Dotplots displayed the smoothed expression distribution of marker genes in distinct CD8^+^ cell types and CD4^+^ cell types.
2. The Ro/e value reflects the enrichment level of T cell subtypes in different tissues.

(C) The bar chart shows the proportion of each cell type in each sample

(D) The proportion of Tc17 cells in different tissue sources.

(E) The box plot shows the proportion of Tc17 cells, Th17 cells, and cytotoxic T cells (CD8_Trm, CD8_Tem, CD8_Teff, CD8_GZMK_ZNF683, CD8_Blood_Teff) in the TCGA deconvolution data (lower) and single-cell data (upper) and their statistical differences. P-values were calculated by wilcox.test. *P < 0.05, **P < 0.01, ***P < 0.001, ****P < 0.0001.

(F) The UMAP display the expression of the regulators RORC, CEBPD and ZNF669 within T cells on the left, and the predicted average activity levels from software analysis on the right.

(G) Top 3 regulons enriched in T cell types associated with normal tissues(right) and an intermediate state between tumor cells and normal cells(left). The darker the color, the stronger the regulatory impact of the modulator.

**Figure S3. Assessing the degree of malignancy of epithelial cells**

(A) The violin plot shows the INFG expression levels of different CD8 subgroups calculated by the pseudobulk method, ranked from highest to lowest. Scatter plots display the intersection of differentially expressed genes (DEGs) specific to Tc17 cells when compared to other CD8^+^ Cytotoxic T cells. Bar charts indicate the pathways enriched by the two sets of genes.

(B) The copy number alterations of malignant epithelial cells inferred by inferCNV. T&NK cells and endocrine cells were selected as normal reference, and the copy number gain was shown in red color and the copy number loss were shown in blue color.

(C) Expression of top10 differential genes among different subpopulations of epithelial cells.

(D) GSEA outcomes indicate the enrichment of Cell Cycle and Glycerophospholipid Metabolism gene sets in malignant epithelial cells, while normal epithelial cells show enrichment in Activation of Immune Response and immune response-regulating signaling pathway gene sets.

(E) Association of the number of Tc17 cells per sample with tumor subpopulation1,2,4.

**Figure S4. The interactions between tumor cells and T cells**

(A) The interaction of CXCL pathway in the tumor system, the arrow from the ligand to the receptor, the width represents the intensity of the interaction.

(B) The expression of significant interactions between tumor cells and T cells in the tumor system.

(C) The expression levels of IL16 and IL17A were correlated in TCGA data.

(D) All interleukin levels expressed by T cell subpopulations in the tumor system.

**Figure S5. Spatial Transcriptomic Data Confirms the Proximity of Tumor Cells and Tc17 Cells**

(A) The bar chart illustrates the proportion of Tc17 cells surrounding tumor regions (In the same spot) and non-tumor regions..

(B) The spatial expression of cell type-specific markers of CD8 T cells (CD3D, CD3E, CD8A and CD8B), Tc17 cells (IL17A and RORC), tumor cells (KRT17).

(C) The boxplot shows the distance between tumor cells and the nearest Tc17 cells, as well as the distance between non-tumor cells and the nearest Tc17 cells.

(D) The boxplot illustrates the average expression of CXCR6 in Tc17 cells surrounding CXCL16^+^ tumor cells and CXCL16^−^ cells.


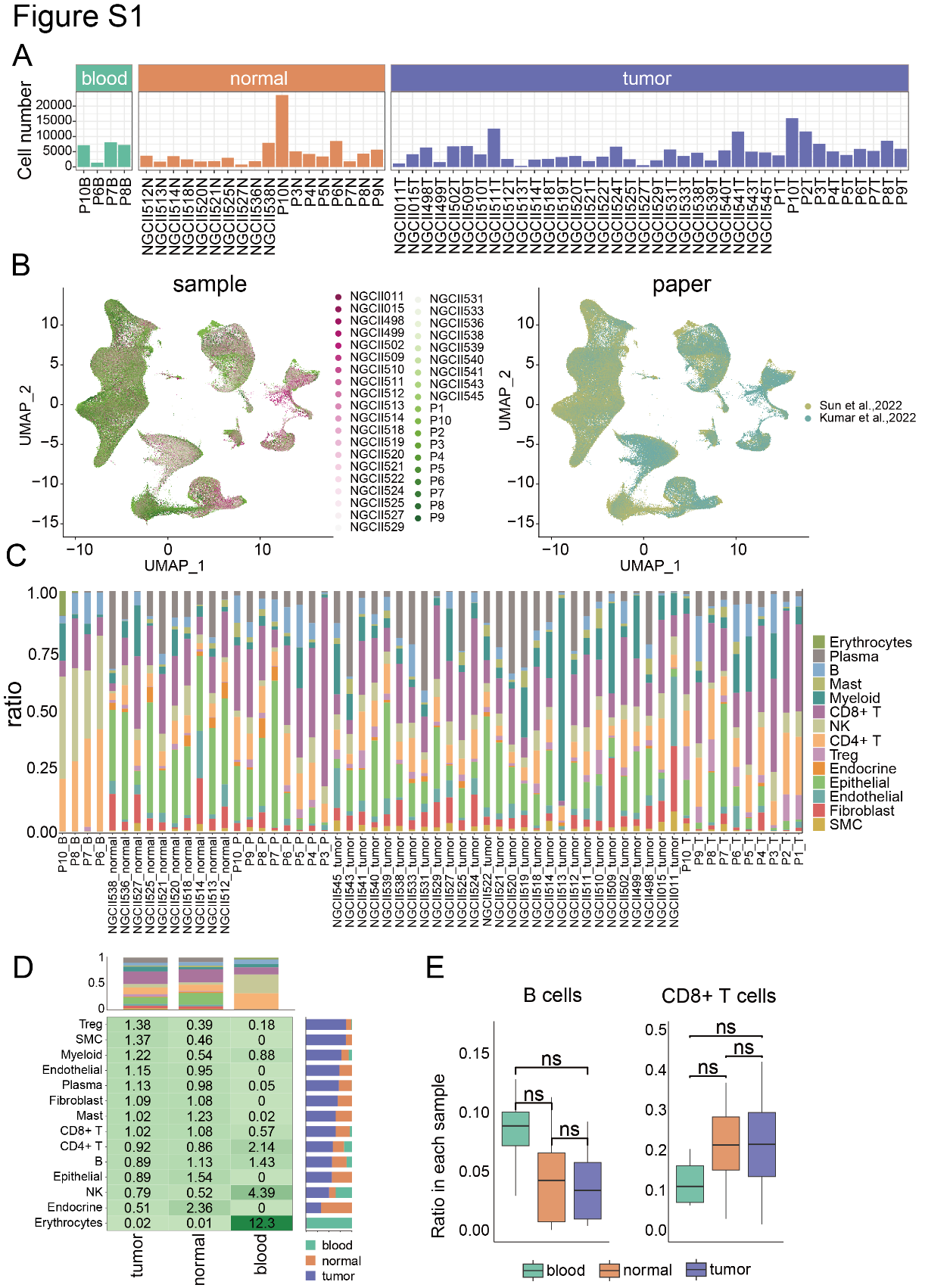


**Figure S1. Dynamic alterations in cell populations within the tumor microenvironment**


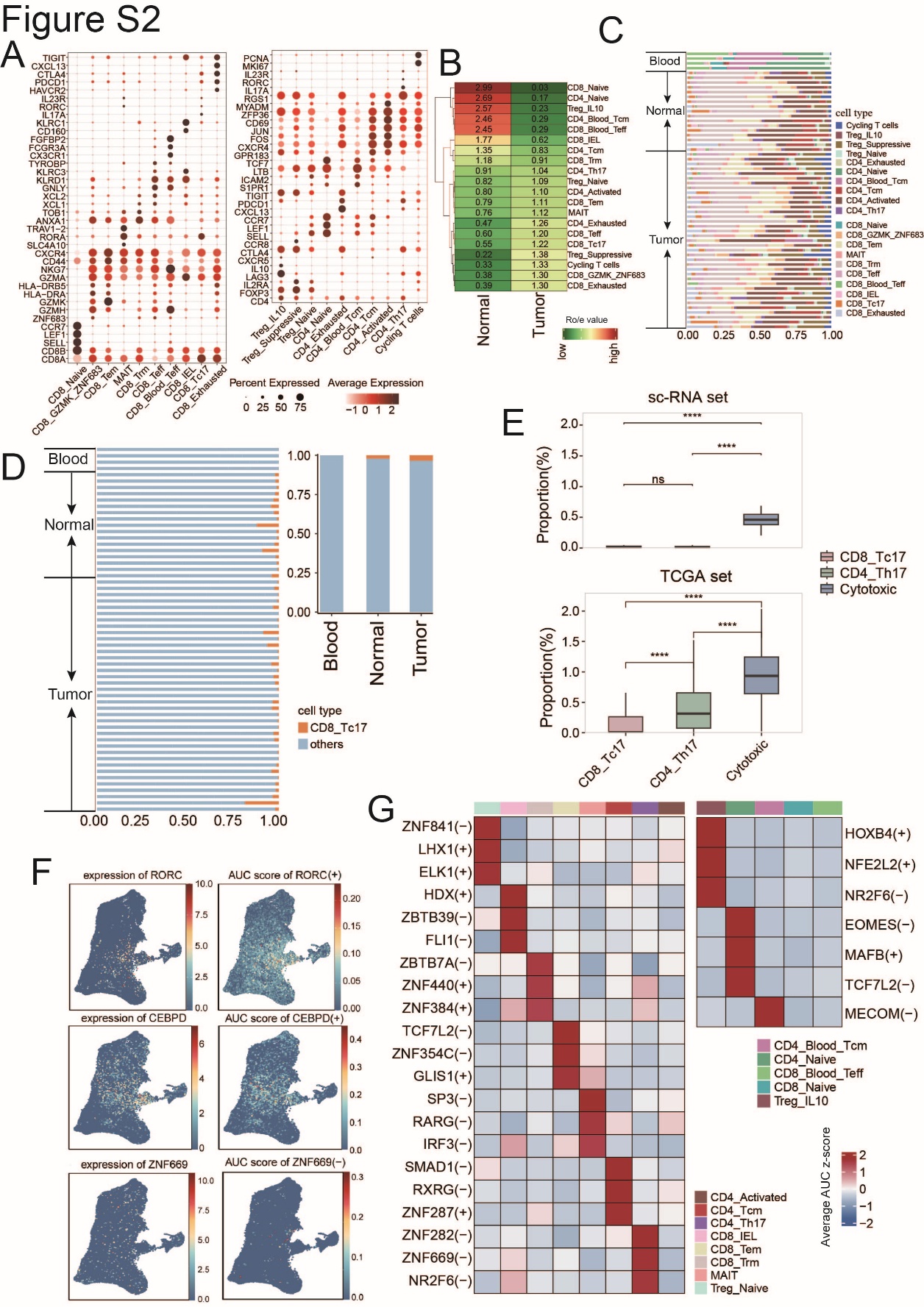


**Figure S2. The profiling of T cell subsets**


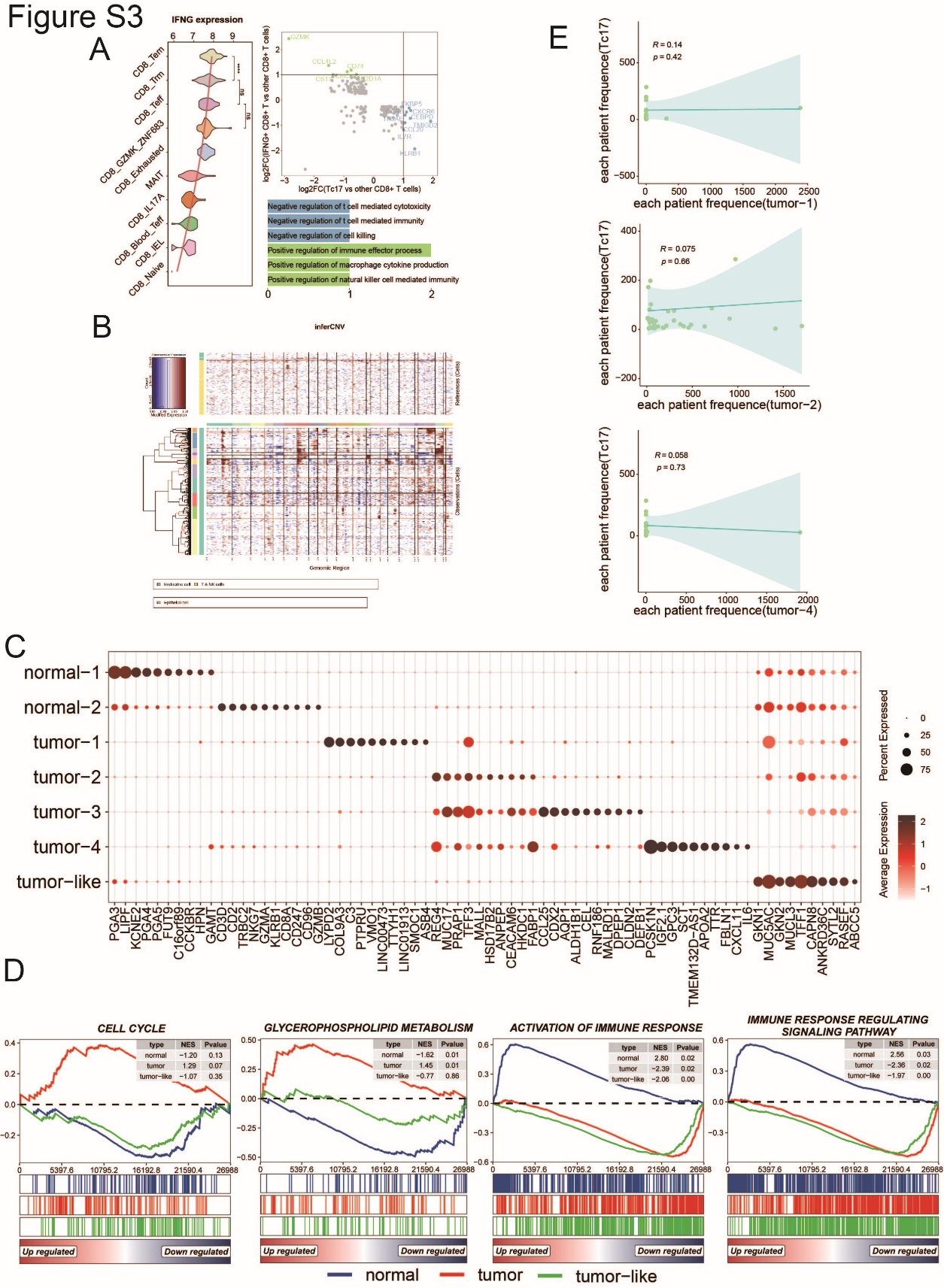


**Figure S3. Assessing the degree of malignancy of epithelial cells**


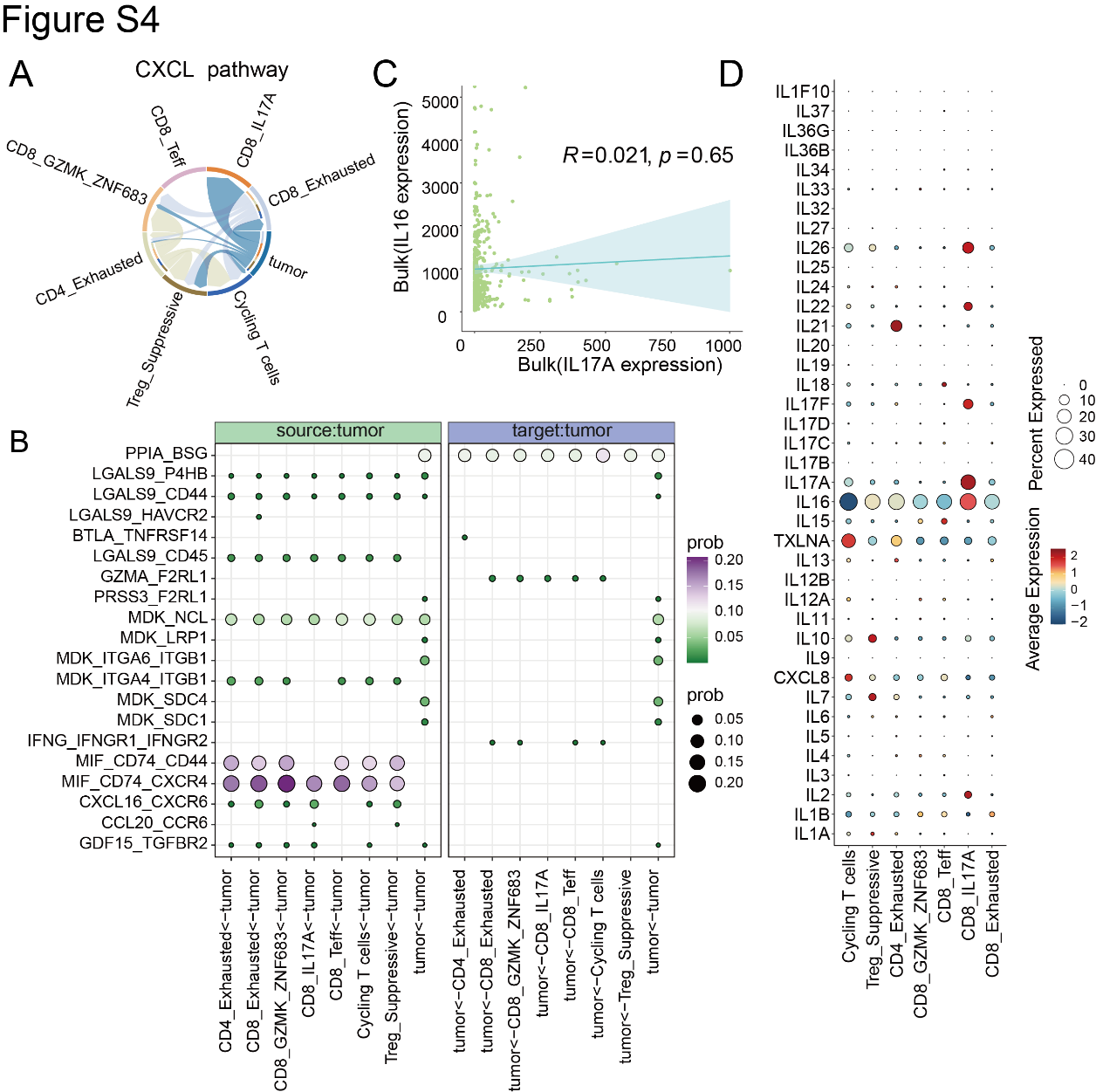


**Figure S4. The interactions between tumor cells and T cells**


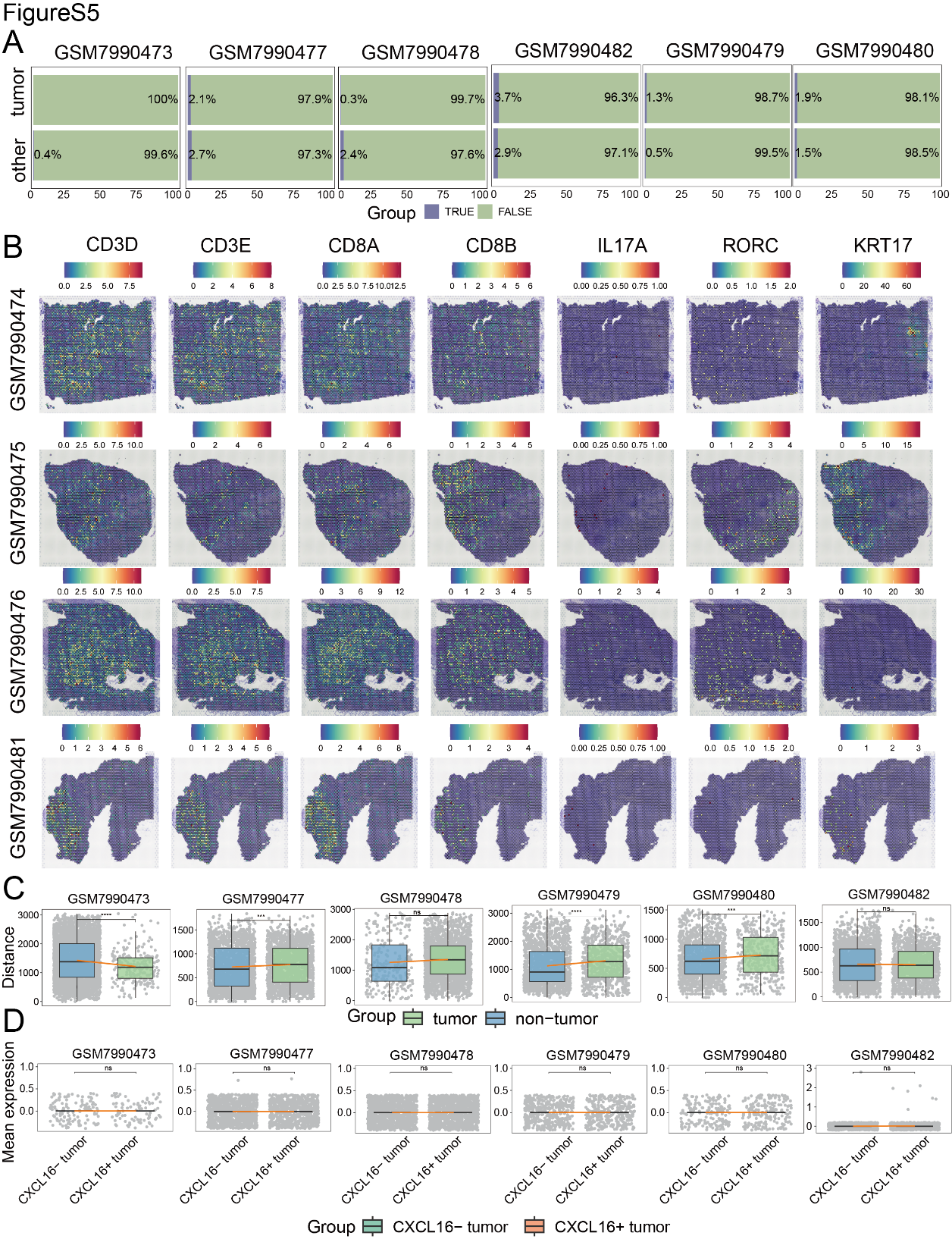


**Figure S5. Spatial Transcriptomic Data Confirms the Proximity of Tumor Cells and Tc17 Cells**
